# *p*‐Terphenyl and Orsellinic Acid Derivatives from the European Polyporales *Terana coerulea* and *Sparassis brevipes*

**DOI:** 10.1002/cbdv.202401597

**Published:** 2024-11-04

**Authors:** Winnie Chemutai Sum, Sherif S. Ebada, Harald Kellner, Marc Stadler

**Affiliations:** ^1^ Department of Microbial Drugs Helmholtz Centre for Infection Research GmbH (HZI) Inhoffenstraße 7 38124 Braunschweig Germany Tel.; ^2^ Institute of Microbiology Faculty of Life Sciences Institute of Microbiology Technische Universität Braunschweig Spielmannstraße 7 38106 Braunschweig Germany; ^3^ Department of Pharmacognosy Faculty of Pharmacy Ain Shams University 11566 Cairo Egypt; ^4^ Department of Bio- and Environmental Sciences Technische Universität Dresden-International Institute Zittau Markt 23 02763 Zittau Germany

**Keywords:** *Corticium caeruleum*, *Sparassis brevipes*, Corticin, Orsellinic acid, Lichen depsides

## Abstract

Investigation of the secondary metabolites of the solid‐state rice cultures from the European basidiomycetes *Terana coerulea* and *Sparassis brevipes* afforded three previously undescribed secondary metabolites identified as two *p*‐terphenyl derivatives (**1** and **2**) and one orsellinic acid congener (**3**) in addition to another known, isoeverninic acid (**4**). Chemical structures of the isolated compounds were elucidated through comprehensive 1D and 2D NMR spectroscopic analyses, coupled with HR‐MS and other spectral methods. The isolated compounds were assessed for their cytotoxic and antimicrobial activities. Compound **1** revealed weak antimicrobial properties, whereas the inseparable mixture of **3** and **4** featured moderate cytotoxic and antimicrobial effects.

## Graphical abstract

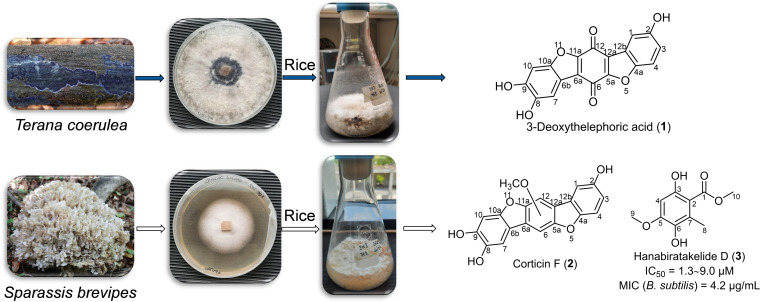

## 1 Introduction

Forest ecosystems rely on polypores for regeneration and energy recycling as pathogens, wood‐degraders and ectomycorrhizal symbionts. Apart from their ecological significance, basidiocarps of some polypores also serve as a human food and a source of medicines. A recent report revealed that the tropics harbor a huge number of polypore species compared to the boreal and temperate regions, with the orders Polyporales and Hymenochaetales accounting for 93.4 % of the reported species.^[1]^

The order Polyporales to which the current study fungi belong, consists of about 1,800 saprotrophic species of the division Basidiomycota. The cobalt crust fungus *Terana coerulea* (formerly *Corticium caeruleum*, Phanerochaetaceae), was reported to be used locally as an antibiotic in Spain.^[2]^ In that study, unprecedented cytotoxic molecules named corticins D–E in addition to corticin A were isolated from the dry fruiting bodies.^[2]^ In addition, earlier studies of the fungus led to the discovery of similar bioactive corticin analogues and reported its genome.[^3^, ^4^, ^5^] On the other hand, the edible and rare basidiomycete *Sparassis brevipes* (Sparassidaceae) remains hitherto unexplored for its secondary metabolites.

In our continued exploration of the seldom‐found Basidiomycota of Western Europe for novel chemistry, we encountered the rare polypores *T. coerulea* and *S. brevipes* (latter appearing on the Red List of Germany). Herein, we report the structure elucidation of previously undescribed compounds, as well as the results of bioassays.

## 2 Results and Discussion

### 2.1 Strain Identification

Both fungal specimens were collected and morphological identified by one of the authors (H.K.). *Terana coerulea* IHI769 was collected from a lying *Fraxinus excelsior* log, on the 4^th^ September 2022 in a mixed deciduous forest at Wiesbaden‐Bierstadt (Germany). *Sparassis brevipes* IHI752 was collected at the base of an *Abies alba* tree, on the 19^th^ August 2021 in a near natural forest (Mittelsteighütte) close to the Zwieseler Waldhaus in the Bavarian Forest National Park, Germany. Generally, clean small pieces of the fruiting bodies were cut and placed on agar plates containing malt medium with antibiotics or just water agar, thus isolated and later subcultured (see below). From both isolates the ITS‐rRNA was also amplified, sequenced and submitted to GenBank with the accession codes PP957925 (*T. coerulea* IHI769) and PP957926 (*S. brevipes* IHI752).

### 2.2 Structure Elucidation of Compounds 1–4

**Figure 1:**
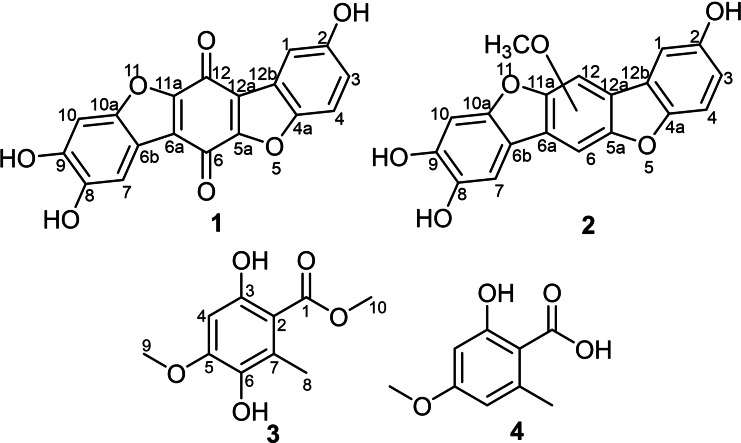
Chemical structures of **1**–**4**.

The individual solid‐state rice culture extracts of the two European polypores *T. coerulea* and *S. brevipes* were extracted with EtOAc and the afforded extracts were subjected to dereplication study applying HPLC‐DAD‐MS. The revealed results were compared to the reported literature in secondary metabolite databases, (), and exhibited the presence of previously undescribed and known compounds (Figure 1). Therefore, the crude organic extracts were purified using preparative HPLC techniques that yielded two previously undescribed *p*‐terphenyl metabolites (**1** and **2**) (Figure 1) from *T. coerulea* and *S. brevipes*, respectively. In addition, an inseparable mixture of one undescribed and another known natural orsellinic acid derivatives (**3** and **4**) were obtained from *S. brevipes* extract.

**Figure 2:**
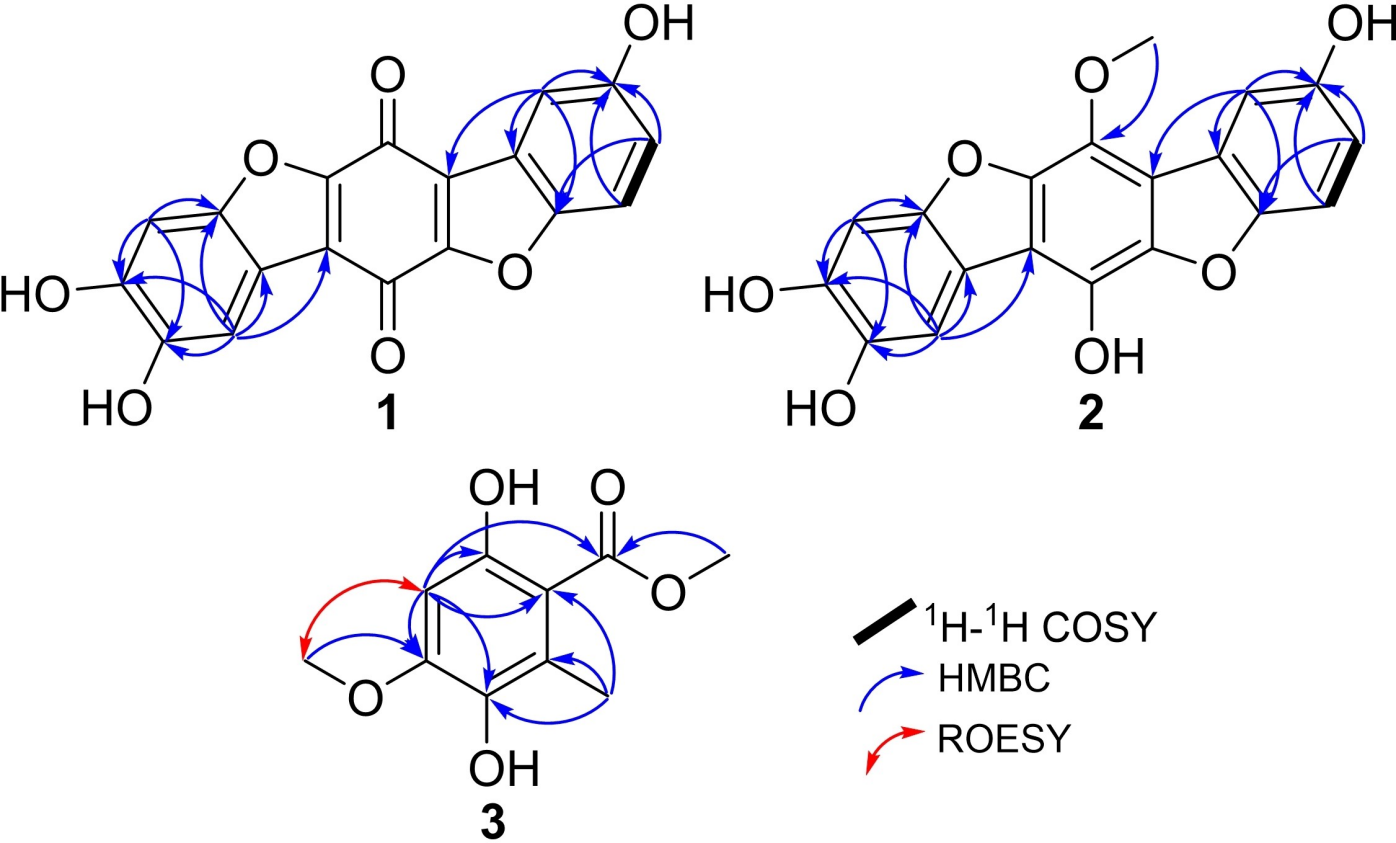
Key ^1^H−^1^H COSY, HMBC and ROESY correlations of **1–3**.

Compound **1** was obtained as a brown solid powder. Its molecular formula was determined to be C_18_H_8_O_7_, indicating fifteen degrees of unsaturation based on HR‐ESI‐MS that revealed a protonated molecule at *m/z* 337.0345 [M+H]^+^ (calculated 337.0343). The high degrees of unsaturation, compared to the relatively small molecular weight, suggested the presence of a highly conjugated chromophore that was further supported by the UV spectrum of **1** (Figure S2) revealing several absorption maxima (λ_max_) over the UV‐Vis range at 201, 224, 258, 291, 450 nm. The ^1^H‐NMR, ^1^H−^1^H COSY and HSQC spectra of **1** (Table 1 and Figures 2, S6–S8) revealed the presence of five aromatic proton signals (δ_H_ 6.70~7.38). Three proton signals at δ_H_ 6.70 (*dd*, *J*=8.0, 2.4 Hz, H‐3), 6.82 (*d*, *J*=8.0 Hz, H‐4) and 6.81 (*d*, *J*=2.4 Hz, H‐1) revealed a spin system in its ^1^H−^1^H COSY spectrum (Figure 2) and were directly correlated via HSQC spectrum (Figure S8) to three aromatic carbon atoms at δ_C_ 124.2 (C‐3), 115.9 (C‐4) and 119.3 (C‐1), respectively, indicating the presence of 1,4,5‐trisubstituted aromatic ring. The other two singlet proton signals revealed common key correlations in the HMBC spectrum (Figures 2 and S7) to three oxygenated aromatic carbon atoms at δ_C_ 152.2 (C‐8), 150.8 (C‐9) and 147.6 (C‐10a) indicating their presence on a 1,2,4,5‐tetrasubstituted aromatic ring. A literature search of compound **1** revealed its related structure to thelephoric acid, *p*‐terphenyl compound previously reported from *Polyozellus multiplex* ^[6]^ and *Corticium caeruleum*.^[7]^

**Table 1:** 1D (^1^H and ^13^C) NMR data of **1** and **2**.

	1		2	
pos.	δ_C_,^*a*,*c*^ type	δ_H_^*b*^ (multi, *J*[Hz])	δ_C_,^*a*,*c*^ type	δ_H_^*b*^ (multi, *J*[Hz])
1	119.3, CH	6.81 (d, 2.4)	118.9, CH	6.81 (d, 2.0)
2	145.9, C		146.8, C	
3	124.2, CH	6.70 (dd, 8.0, 2.4)	123.8, CH	6.70 (dd, 8.0, 2.0)
4	115.9, CH	6.82 (d, 8.0)	115.4, CH	6.82 (d, 8.0)
4a	147.3, C		145.5, C	
5a	153.6, C		n.d.	
6	178.4, CO		n.d.*	
6a	122.2, C		121.8, C	
6b	115.4, C		115.1, C	
7	106.6, CH	7.38 (s)	106.2, CH	7.39 (s)
8	152.2, C		153.2, C	
9	150.8, C		150.4, C	
10	99.3, CH	7.04 (s)	98.8, CH	7.05 (s)
10a	147.6, C		147.2, C	
11a	157.1, C		n.d.	
12	181.6, CO		156.8, C*	
12a	130.2, C		123.8, C	
12b	123.0, C		129.8, C	
13			61.5, CH_3_	3.79 (s)

Measured in methanol‐*d*_4_ at [a] 125 MHz for ^13^C and [b] 500 MHz for ^1^H. [c] Assignment confirmed by HMBC and HSQC spectra. * Values could be exchanged within the same column.

By comparing the ^1^H and ^13^C NMR data of **1** to those reported for thelephoric acid and corticins, compound **1** was deduced to lack one oxygen atom compared to thelephoric acid while it revealed a quinone moiety as indicated by the two carbonyl carbons at δ_C_ 178.4 (C‐6) and 181.6 (C‐12) rather than an aromatic ring as in corticins. Further confirmation to the depicted structure of **1** (Figure 1) was obtained by its HMBC spectrum (Figure 2) that revealed key correlations from an aromatic proton assigned as H‐1 to C‐12a (δ_C_ 130.2) and C‐12b (δ_C_ 123.0). In addition, the HMBC spectrum also revealed key correlations of H‐1 and H‐3 to C‐4a (δ_C_ 147.3). Based on the obtained results, compound **1** was confirmed to be a previously undescribed *p*‐terphenyl derivative that was trivially named 3‐deoxythelephoric acid.

Compound **2** was purified as a brown solid powder. The UV spectrum of **2** (Figure S9) revealed three absorption maxima (λ_max_) at 217, 261 and 297 nm overlapping with those of **1** suggesting a related chromophore. However, neither LR‐ nor HR‐ESI‐MS revealed a clear molecular ion peak that could be interpreted as previously reported for related derivatives.^[6]^ The ^1^H‐NMR and HSQC spectra of **2** (Table 1 and Figure S15) revealed five aromatic proton signals at δ_H_ 6.70~7.39 comparable to those revealed in **1**. However, an additional oxygenated methyl group at δ_H_ 3.79, directly correlated to a carbon resonance at δ_H_ 61.5 (C‐13), was recognized. The ^1^H−^1^H COSY spectrum of **2** (Figure 2) revealed a spin system between three aromatic proton signals at δ_H_ 6.70 (*dd*, *J*=8.0, 2.0 Hz, H‐3), 6.82 (*d*, *J*=8.0 Hz, H‐4) and 6.81 (*d*, *J*=2.0 Hz, H‐1) that were directly correlated via HSQC spectrum (Figure S15) to three aromatic carbon atoms at δ_C_ 123.8 (C‐3), 115.4 (C‐4) and 118.9 (C‐1), respectively, indicating the presence of 1,4,5‐trisubstituted aromatic ring. The HMBC spectrum of **2** (Figures 2 and S14) revealed comparable key correlations to those noticed for **1** apart from a clear correlation from the oxygenated methyl group at δ_H_ 3.79 (δ_C_ 61.5) to an oxygenated olefinic carbon at δ_C_ 156.8 (C‐12).

A literature search of **2** revealed its structural resemblance to corticin A, a benzobisbenzofuranoid metabolite first reported from the same fungus (referred to under the synonym *Corticium caeruleum*) in 1976.^[4]^ A comparison of the 1D (^1^H and ^13^C) NMR spectral data of **2** (Table 1) and those reported for corticins A and E^[2]^ revealed a close coherence, with two main differences in having two asymmetrically rather than symmetrically substituted aromatic rings and one methoxy group on *p*‐hyroxyphenol ring in **2** instead of two groups in corticins A and E.^[2]^ Due to the shortage of the obtained amount, the 2D NMR spectrum of **2** in particular ROESY spectrum (Figure S16) could not provide a clear evidence to allocate the methoxy group (OMe‐13) in **2** either at C‐6 or C‐12 and hence remained unassigned. Based on the aforementioned results, compound **2** was deduced to be a previously undescribed neolignan derivative, featuring benzobisbenzofuranoid skeleton reported for corticins,[^2^, ^4^] and was trivially named corticin F.

Compound **3** was purified as a white amorphous solid in an inseparable mixture with isoeverninic acid (5‐*O*‐methylorsellinic acid, **4**).[^8^, ^9^] The 1D (^1^H and ^13^C) NMR spectra of **3** and **4** (Figures S17 and S18) clearly revealed the presence of two sets of signals in an ratio of 5 : 1 based on their integration indices, respectively. The ^1^H‐NMR spectral data of **3** (Table 2) revealed the presence of an aromatic singlet proton signal at δ_H_ 6.37 (H‐4) in addition to three singlet methyl groups, categorized into one aromatic methyl at δ_H_ 2.37 (H_3_‐8) and two methoxy groups at δ_H_ 3.87 (H_3_‐9) and 3.90 (H_3_‐10). The ^13^C NMR and HSQC spectral data of **3** (Table 2 and Figures S18 and S21) revealed ten carbon resonances divided into one carboxylic acid carbon at δ_C_ 173.6 (C‐1), five unprotonated sp^2^ carbon atoms (δ_C_ 158.1, 154.3, 138.8, 126.2, 106.9), one sp^2^ methine at δ_C_ 98.3 (C‐4) and three sp^3^ methyl carbon atoms (δ_C_ 56.5, 52.3, 14.6). The HMBC spectrum of **3** (Figures 2 and S20) revealed key correlations from one methoxy group (H_3_‐10) to C‐1 indicating its presence as a methyl ester moiety. The HMBC spectrum of **3** (Figures 2 and S20) also revealed correlation from one methoxy group (H_3_‐9) to an oxygenated sp^2^ carbon at δ_C_ 154.3 (C‐5) in addition to key correlations from H‐4 to C‐2 (δ_C_ 106.9), C‐3 (δ_C_ 158.1), C‐5, C‐6 (δ_C_ 138.8) along with a long‐range correlation to C‐1; and from H_3_‐8 to C‐2, C‐6 and C‐7 (δ_C_ 126.2) confirming their positions as depicted in Figure 1. A literature search of **3** featuring a trisubstituted methyl benzoate (Figure 1) revealed its structural correlation to orsellinic acid in particular to *β*‐resorcylic acid, an antiproliferartive lichen acid derivative previously reported from *Thamnolia vermicularis* ^[9]^ and hanabiratakelides A–C, phthalide metabolites previously reported from the fruiting bodies of *Sparassis crispa*.^[10]^ Further confirmation to the chemical structure of **3** was provided by the ROESY spectrum (Figure 2) that revealed key correlation from H‐4 to H_3_‐9. Accordingly, compound **3** was concluded to a previously undescribed natural orsellinic acid derivative, methyl 5‐*O*‐methyl‐6‐hydroxylorsellinate that was given a trivial name hanabiratakelide D. It is worth mentioning that compound **3** was only reported in literature as an intermediate in the chemical synthesis of lichen depsides.[^11^, ^12^] To the best of our knowledge, this study unprecedentedly reports it as a natural derivative.

**Table 2:** 1D (^1^H and ^13^C) NMR data of **3**.

pos.	δ_C_,^*a*,*c*^ type	δ_H_^*b*^ (multi, *J*[Hz])
1	173.6, CO	
2	106.9, C	
3	158.1, C	
4	98.3, CH	6.37 (s)
5	154.3, C	
6	138.8, C	
7	126.2, C	
8	14.6, CH_3_	2.37 (s)
9	52.3, CH_3_	3.87 (s)
10	56.5, CH_3_	3.90 (s)

Measured in methanol‐*d*_4_ at [a] 125 MHz for ^13^C and [b] 500 MHz for ^1^H. [c] Assignment confirmed by HMBC and HSQC spectra.

### 2.3 Biological Activity of Compounds 1–4

The isolated compounds were tested in our standardized antimicrobial and cytotoxic assays. 3‐Deoxythelephoric acid (**1**) was non‐cytotoxic and weakly antimicrobial against three of the tested pathogens at 66.6 μg/mL (Table 3). The inseparable mixture of **3** and **4** was cytotoxic against all tested mammalian cell lines at IC_50_ ranging from 1.3–9.4 μM. Similarly, compounds **3** and **4** were antagonistic against some test pathogens; strong effects were recorded against *Bacillus subtilis* (4.2 μg/mL) and *Chromobacterium violaceum* (16.6 μg/mL), moderate antagonism visualized against *Staphylococcus aureus* and *Mucor hiemalis* at 33.3 μg/mL and weak antimicrobial effects against *Acinetobacter baumannii*, *Mycobacterium smegmatis*, *Schizosaccharomyces pombe*, *Candida albicans* and *Rhodotorula glutinis* at 66.6 μg/mL. Compound **2** revealed no significant activity in either bioassays (Table 3).

**Table 3:** Cytotoxicity (IC_50_) and antimicrobial activity (MIC) of **1**–**4**.

	IC_50_ (μM)			Positive Control
Test Cell Line	1	2	3/4	Epothilone B (nM)
Mouse fibroblast (L929)	68.5	n.a.	1.3	0.65
Human endocervical adenocarcinoma (KB3.1)	80.4	n.a.	3.2	0.17
Human prostate carcinoma (PC‐3)	n.d.	n.d.	8.0	0.09
Human breast adenocarcinoma (MCF‐7)	n.d.	n.d.	2.0	0.07
Human ovarian cancer (SKOV‐3)	n.d.	n.d.	6.6	0.09
Human epidermoid carcinoma (A431)	n.d.	n.d.	n.d.	0.06
Human lung carcinoma (A549)	n.d.	n.d.	9.4	0.05

Test Microorganism	MIC (μg/mL)			Positive Control (μg/mL)
*Staphylococcus aureus*	66.6	n.i.	33.3	0.42^G^
*Escherichia coli*	n.i.	n.i.	n.i.	0.83^G^
*Bacillus subtilis*	66.6	n.i.	4.2	16.6^O^
*Pseudomonas aeruginosa*	n.i.	n.i.	n.i.	0.42^G^
*Pichia anomala*	n.d.	n.i.	n.i.	8.30^N^
*Candida albicans*	n.d.	n.i.	66.6	8.30^N^
*Acinetobacter baumannii*	n.i.	n.i.	66.6	1.04^C^
*Chromobacterium violaceum*	n.i.	n.i.	16.6	1.67^G^
*Schizosaccharomyces pombe*	n.d.	n.i.	66.6	8.30^N^
*Mucor hiemalis*	66.6	n.i.	33.3	8.30^N^
*Rhodotorula glutinis*	n.d.	n.i.	66.6	4.20^N^
*Mycobacterium smegmatis*	n.d.	n.i.	66.6	1.70^K^

n.a.: No activity. n.i.: No inhibition up to 67 μg/mL. n.d.: Not determined. G: Gentamicin; O: Oxytetracycline; N: Nystatin; C: Ciprofloxacin; K: Kanamycin.

According to the reported literature, orsellinic acid esters revealed significant cytotoxic activities against several cell lines rather than its free acid form^[13]^ and hence supports a principal role of hanabiratakelide D (**3**) rather than isoeverninic acid (**4**) as a cytotoxic agent. In addition, methyl 3‐methylorsellinate was reported in literature to possess potent antitubercular activity with a minimum inhibitory concentration (MIC) of 3.9 μg/mL^[14]^ that also supports the potential activity of orsellinic acid esters as antimicrobial agents.

## 3 Conclusions

In this study, we investigated the extracts of two European polyporales namely *Terana coerulea* and *Sparassis brevipes* for novel bioactive secondary metabolites (SMs). The research outcomes unveiled two previously undescribed *p*‐terphenyl derivatives namely 3‐deoxythelephoric acid (**1**) and corticin F (**2**) from *Terana coerulea* and *Sparassis brevipes*, respectively, along with hanabiratakelide D (**3**) and isoeverninic acid (**4**) from the latter. The inseparable mixture of **3** and **4** proved highly cytotoxic but moderately antimicrobial activities, whereas **1** was weakly antimicrobial. Similarly, hanabiraketalides A–C isolated from *S. crispa* had previously demonstrated strong anticancer effects in addition to their antioxidant and anti‐inflammatory activities.^[10]^ Thus, this class of compounds might be considered as chemotaxonomic marker metabolites of the genus. Previously, highly methoxylated terphenyl derivatives were shown to be cytotoxic.^[2]^ Therefore, the presence methoxy groups could be responsible for inducing cytotoxicity to some extent, since their absence in 3‐deoxythelephoric acid (**1**) and corticin F (**2**) rendered them non‐cytotoxic. Nonetheless, structure‐activity relationship (SAR) studies of these derivatives merit further investigations.

## Materials and Methods

### General Experimental Procedures

UV/Vis spectra were measured on Shimadzu UV/Vis 2450 spectrophotometer (Kyoto, Japan) at a concentration of 0.02 mg/mL in methanol Uvasol^®^ (Merck, Darmstadt, Germany). The Avance III 500 (^1^H: 500 MHz, ^13^C: 125 MHz, Bruker Daltonics, Bremen, Germany) spectrometer was used to record 1D and 2D NMR spectra of the compounds dissolved in deuterated methanol. Chemical shifts were recorded in parts per million (ppm) and coupling constants were calculated in Hertz (Hz). HPLC‐DAD/MS analyses were performed on the amaZon speed ETD ion trap mass spectrometer (Bruker Daltonics, Bremen, Germany), on both positive and negative ionization modes. The HPLC system consisted of a Dionex UltiMate 3000 UHPLC (Thermo‐Fisher Scientific Inc., Waltham, MA, USA) equipped with a C_18_ Acquity UPLC BEH column (Waters, Milford, USA). The solvent phase consisted of solvent A: deionized H_2_O+0.1 % formic acid (FA) and solvent B: acetonitrile (MeCN)+0.1 % FA. The separation gradient was as follows: 5 % of solvent B for 0.5 min, 5–100 % solvent B over 20 min and holding the gradient isocratically at 100 % solvent B for 10 min. The flow rate employed was 0.6 mL/min and the UV/Vis detections recorded at 190–600 nm. HR‐(+)ESI‐MS data were recorded on a maXis ESI‐TOF (Time‐Of‐Flight) mass spectrometer (Bruker Daltonics, Bremen, Germany) connected to an Agilent 1260 series HPLC‐UV system (Agilent Technologies, Santa Clara, CA, USA) equipped with a C_18_ Acquity UPLC BEH column (Waters). The solvent system consisted of solvent A (deionized H_2_O+0.1 % FA) and solvent B (MeCN+0.1 % FA). The separation gradient was as follows: 5 % solvent B for 0.5 min, 5–100 % solvent B over a period of 19.5 min and eventually holding solvent B at 100 % for 5 min. The flow rate employed was 0.6 mL/min at 40 °C and the UV/Vis detection recorded at 200–600 nm. Molecular formulas of the detected compounds were calculated using the Smart Formula algorithm of the Compass Data Analysis software (Bruker, version 6.1).

The solvents and chemicals (analytical and HPLC grades) used, were sourced from Merck (Darmstadt, Germany), Avantor Performance Materials (Deventer, Netherlands), AppliChem GmbH (Darmstadt, Germany) and Carl Roth GmbH & Co. KG (Karlsruhe, Germany). Deionized water was prepared using the Purelab^®^ flex water purification system (Veolia Water Technologies, Celle, Germany).

### Collection of the Fungal Specimen and Preparation of Cultures

The specimens were collected and identified by one of the authors (H.K.). After first isolation on water agar or malt agar plates containing antibiotics, the mycelial cultures were cultured on YMG (4 g of yeast extract, 10 g of malt extract, 4 g of glucose, and 20 g of agar in 1 L of deionized water) medium, and copies deposited at the International Institute Zittau, Technical University of Dresden (Germany) as IHI 769 (*Terana coerulea*) and IHI 752 (*Sparassis brevipes*).

### Fermentation of Cultures and Extraction of Metabolites

Fully grown axenic mycelial cultures of the two fungal strains maintained on YMG agar plates were used to inoculate 5×500 mL Erlenmeyer culture flasks containing sterile rice medium, for each fungal strain as previously described.^[15]^ After 36 and 48 days for *T. coerulea* and *S. brevipes*, respectively, the fermented cultures were extracted. Initially, the cultures were soaked overnight in 3×500 mL of acetone per flask under sonication at 40 °C for 45 min. The solvent was evaporated after filtration and the attained aqueous phase was partitioned twice against EtOAc (1 : 1). The aqueous phase was discarded and the EtOAc portion was dried under reduced pressure using a rotary evaporator to yield crude extracts of 2.0 g (*T. coerulea*) and 1.0 g (*S. brevipes*).

### Isolation of Compounds and Their Physico‐Chemical Properties

The afforded EtOAc extracts were purified on preparative HPLC (PLC 2020; Gilson, Middleton, WI) system, using the C_18_ VP‐Nucleodur column 100–5 (250×40 mm, 7 μm: Macherey‐Nagel, Düren, Germany) as the stationary phase. The solvent system consisted of deionized H_2_O+0.1 % formic acid (FA) as solvent A and MeCN+0.1 % FA as solvent B. The flow rate was set at 40 mL/min and the UV detection was made at 190, 210, and 280 nm wavelengths.

The gradient implemented to elute the compounds was as follows: an initial isocratic condition of 5 % solvent B for 10 min, followed by an increase from 5–15 % of solvent B within 5 min. Thereafter, a gradient increase from 15–45 % of solvent B took place over 40 min, 45–100 % within 10 min and a final holding of the gradient at 100 % of solvent B for 10 min. The procedure led to the isolation of **1** (1.0 mg, *t*_R_=23 min), **2** (0.5 mg, *t*_R_=20 min) and **3**/**4** (1.3 mg, *t*_R_=30 min).

*3‐Deoxythelephoric acid (**1**)*. Brown solid powder; UV/Vis (MeOH): λ_max_=201, 224, 258, 291, 450 nm; NMR data (^1^H: 500 MHz, ^13^C: 125 MHz in methanol‐*d*_4_) see Table 1; HR‐ESI‐MS: *m/z* 337.0345 [M+H]^+^ (calcd. 337.0343 for C_18_H_9_O_7_^+^), 369.0607 [M+CH_3_OH+H]^+^ (calcd. 369.0605 for C_19_H_13_O_8_^+^); *t*_R_=5.74 min (LR‐ESI‐MS). C_18_H_8_O_7_ (336.11 g/mol).

*Corticin F (**2**)*. Brown solid poweder; UV/Vis (MeOH): λ_max_=217, 261, 297 nm; NMR data (^1^H: 500 MHz, ^13^C: 125 MHz in methanol‐*d*_4_) see Table 1; *t*_R_=3.21 min (LR‐ESI‐MS). C_19_H_12_O_7_ (352.31 g/mol).

*Hanabiratakelide D (**3**)*. Off‐white amorphous solid; UV*/*Vis (MeOH): λ_max_=226 nm; NMR data (^1^H: 500 MHz, ^13^C: 125 MHz in methanol‐*d*_4_) see Table 2; *t*_R_=13.11 min (LR‐ESI‐MS). C_10_H_12_O_5_ (212.20 g/mol).

*Isoeverninic acid (**4**)*. Brown oil; UV/Vis (MeOH): λ_max_=226 nm; NMR data (^1^H: 500 MHz, ^13^C: 125 MHz in methanol‐*d*_4_) see Table S1 compared to the reported literature;[^8^, ^9^] *t*_R_=13.11 min (LR‐ESI‐MS). C_9_H_10_O_4_ (182.18 g/mol).

### Antimicrobial Assay

The antimicrobial effects of the isolated compounds were determined against a panel of twelve different microorganisms applying our established protocols.[^16^, ^17^] The minimum inhibitory concentrations (MIC) were recorded after an overnight incubation, as the lowest concentration under which no microbial growth was visualized. The microorganisms were obtained from the German Collection of Microorganisms and Cell Cultures (DSMZ, Braunschweig, Germany).

### Cytotoxicity (MTT) Assay

MTT (3‐(4,5‐dimethylthiayol‐2‐yl)‐2,5‐diphenyltetrazolium bromide)‐based assay was used to determine *in vitro* cytotoxicity against a panel of seven different cell lines following our previously reported protocol.[^16^, ^17^]

## Author Contributions

W.C.S.: conceptualization, large‐scale fermentation, isolation and structure elucidation of the compounds, antimicrobial assays and preparation of the original draft; S.S.E.: structure elucidation of compounds, editing and polishing the draft; H.K.: isolation and identification of the producer strain; M.S.: supervision, funding acquisition, correcting, editing, and polishing the draft. All authors have read and agreed to the published version of the manuscript.

## Conflict of Interests

The authors declare no conflict of interest.

4

## Supporting information

As a service to our authors and readers, this journal provides supporting information supplied by the authors. Such materials are peer reviewed and may be re‐organized for online delivery, but are not copy‐edited or typeset. Technical support issues arising from supporting information (other than missing files) should be addressed to the authors.

Supporting Information

## Data Availability

The data that support the findings of this study are available in the supplementary material of this article.
