# Supplementary material for: p‐Terphenyl and Orsellinic Acid Derivatives from the European Polyporales Terana coerulea and Sparassis brevipes
Source: Chem Biodivers. 2024 Nov 4;22(1):e202401597. doi: 10.1002/cbdv.202401597 (PMC11741153; doi:10.1002/cbdv.202401597)
Supplement: Supplementary file 1 — Supporting Information [file CBDV-22-e202401597-s001.pdf]

Chemistry & Biodiversity

Supporting Information

p-Terphenyl and Orsellinic Acid Derivatives from the European Polyporales *Terana coerulea* and *Sparassis brevipes*

Winnie Chemutai Sum, Sherif S. Ebada,* Harald Kellner, and Marc Stadler*

Supporting Information for:

p-Terphenyl and Orsellinic Acid Derivatives from European Polyporales *Terana coerulea* and *Sparassis brevipes*

Winnie Chemutai Sum,[†,‡] Sherif S. Ebada,*[†,§] Harald Kellner,[¶] and Marc Stadler*[†, ‡]

[†] Department of Microbial Drugs, Helmholtz Centre for Infection Research GmbH (HZI), Inhoffenstraße 7, 38124 Braunschweig, Germany.

[‡] Institute of Microbiology, Technische Universität Braunschweig, Spielmannstraße 7, 38106 Braunschweig, Germany.

[§] Department of Pharmacognosy, Faculty of Pharmacy, Ain Shams University, 11566 Cairo, Egypt.

[¶] Department of Bio- and Environmental Sciences, Technische Universität Dresden-International Institute Zittau, Markt 23, 02763 Zittau, Germany.

* Correspondence: sherif.elsayed@helmholtz-hzi.de; sherif_elsayed@pharma.asu.edu.eg (S.S.E.); Marc.Stadler@helmholtz-hzi.de (M.S.); Tel.: +49-531-6181-4240; Fax +49-531-6181-9499

Contents of Supporting Information

#	Contents	Page
**1**	Figure S1. Key ^{1}H–^{1}H COSY, HMBC and ROESY correlations of **4**.	**S3**
**2**	Table S1. ^{1}H and ^{13}C NMR data of compound (**4**) and isoeverninic acid.[1,2]	**S3**
**3**	Figure S2. LR-ESI-MS of **1**.	**S4**
**4**	Figure S3. HR-ESI-MS of **1**.	**S5**
**5**	Figure S4. ^{1}H NMR spectrum of **1** in methanol-d_4 at 500 MHz.	**S6**
**6**	Figure S5. ^{13}C NMR spectrum of **1** in methanol-d_4 at 125 MHz.	**S7**
**7**	Figure S6. ^{1}H–^{1}H NMR spectrum of **1** in methanol-d_4 at 500 MHz.	**S8**
**8**	Figure S7. HMBC spectrum of **1** in methanol-d_4 at 500 MHz.	**S9**
**9**	Figure S8. HSQC spectrum of **1** in methanol-d_4 at 500 MHz.	**S10**
**10**	Figure S9. ROESY spectrum of **1** in methanol-d_4 at 500 MHz.	**S11**
**11**	Figure S10. LR-ESI-MS of **2**.	**S12**
**12**	Figure S11. HR-ESI-MS of **2**.	**S13**
**13**	Figure S12. ^{1}H NMR spectrum of **2** in methanol-d_4 at 500 MHz.	**S14**
**14**	Figure S13. ^{1}H-^{1}H COSY spectrum of **2** in methanol-d_4 at 500 MHz.	**S15**
**15**	Figure S14. HMBC spectrum of **2** in methanol-d_4 at 500 MHz.	**S16**
**16**	Figure S15. HSQC spectrum of **2** in methanol-d_4 at 500 MHz.	**S17**
**17**	Figure S16. ROESY spectrum of **2** in methanol-d_4 at 500 MHz.	**S18**
**18**	Figure S17. ^{1}H NMR spectrum of **3** and **4** in methanol-d_4 at 500 MHz.	**S19**
**19**	Figure S18. ^{13}C NMR spectrum of **3** and **4** in methanol-d_4 at 125 MHz.	**S20**
**20**	Figure S19. ^{1}H-^{1}H COSY spectrum of **3** and **4** in methanol-d_4 at 500 MHz.	**S21**
**21**	Figure S20. HMBC spectrum of **3** and **4** in methanol-d_4 at 500 MHz.	**S22**
**22**	Figure S21. HSQC spectrum of **3** and **4** in methanol-d_4 at 500 MHz.	**S23**
**23**	Figure S22. ROESY spectrum of **3** and **4** in methanol-d_4 at 500 MHz.	**S24**

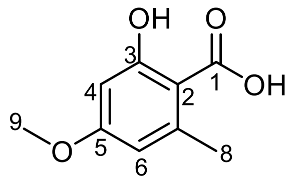

Isoeverninic acid (5-*O*-methylorsellinic acid, **4**)

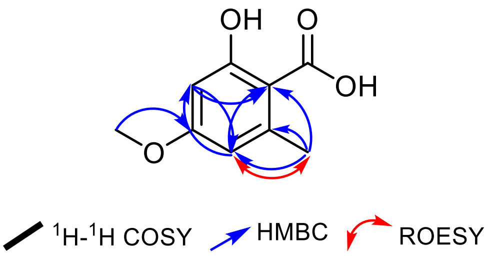

Figure S1. Key ^{1}H–^{1}H COSY, HMBC and ROESY correlations of **4**.

Table S1. ^{1}H and ^{13}C NMR data of compound (**4**) and isoeverninic acid (5-*O*-methylorsellinic acid).[1,2]

	**4**		Isoeverninic acid[1,2]	
**pos.**	**δ_C,^{a,b} type**	**δ_H^{a} (multi, *J*[Hz])**	**δ_C,^{c} type**	**δ_H^{c} (multi, *J*[Hz])**
1	173.6, CO		174.1, CO	
2	105.9, C		105.5, C	
3	166.3, C		167.2, C	
4	101.8, CH	6.15 d (2.5)	99.4, CH	6.33 d (2.5)
5	164.0, C		165.1, C	
6	112.6, CH	6.20 dd (2.5, 1.0)	111.4, CH	6.35 d (2.5)
7	144.7, C		144.5, C	
8	24.4, CH_3	2.44 s	24.3, CH_3	2.57 s
9	52.26, CH_3	3.87 s	55.7, CH_3	3.80 s

[1] Y. Nishitoba, H. Nishimura, T. Nishiyama, J. Mizutani, *Phytochemistry* **1987**, *26*, 3181-3185.
[2] J. Guo, Z.-L. Li, A.-L. Wang, X.-Q. Liu, J. Wang, X. Guo, Y.-K. Jing, H.-M. Hua, *Planta Med.* **2011**, *77*, 2042–2046..

^{a} Measured in methanol-d_4 at 125 MHz for ^{13}C and 500 MHz for ^{1}H.
^{b} Assignment confirmed by HMBC and HSQC spectra.
^{c} Measured in chloroform-*d* at 75 MHz for ^{13}C and 300 MHz for ^{1}H.

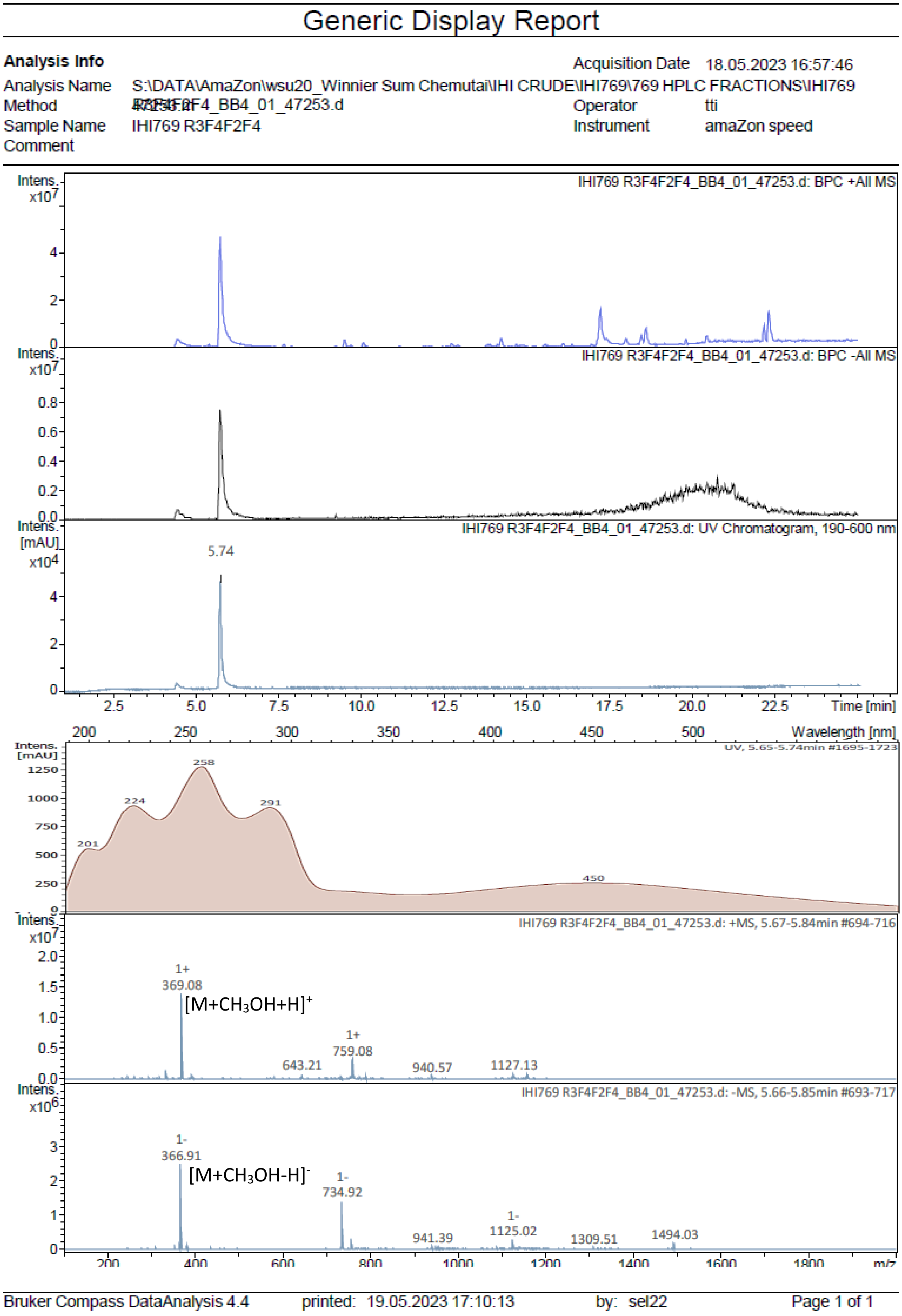

Figure S2. LR-ESI-MS of **1**.

Generic Display Report

**Analysis Info**		Acquisition Date	23.05.2023 18:27:31
Analysis Name	S:\DATA\MaXis\wsu20_Winnie Sum Chemutai\23_05\IHI 769 R3F4F2F4_24_01_11587.d		
Method	pos_säure_10000_screening_ms_100_2500_line.m	Operator	ate06
Sample Name	IHI 769 R3F4F2F4	Instrument	maXis
Comment	Screening01<br>Waters Acquity UPLC BEH C_18 1,7um 2.1x50mm		

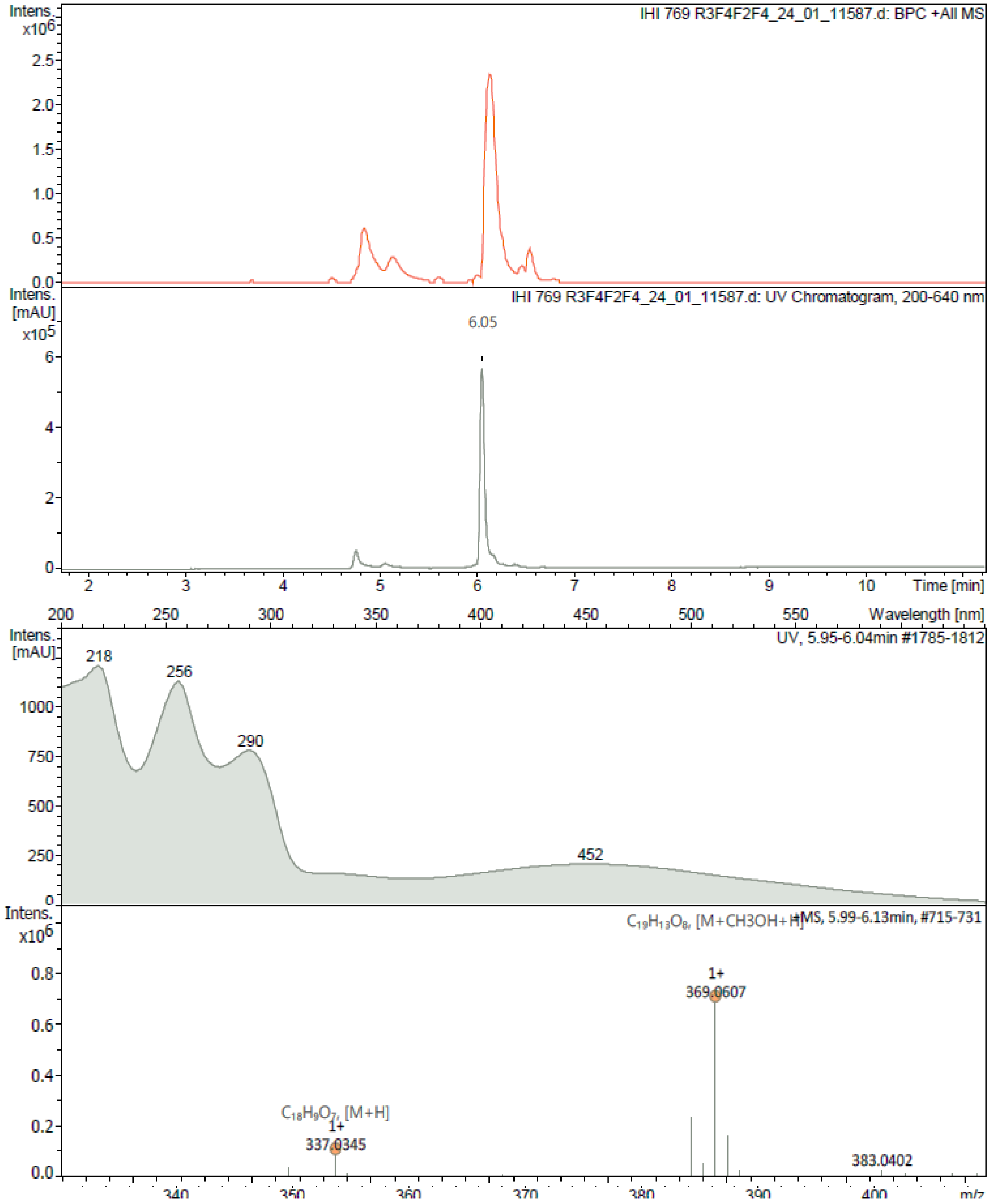

Figure S3. HR-ESI-MS of **1**.

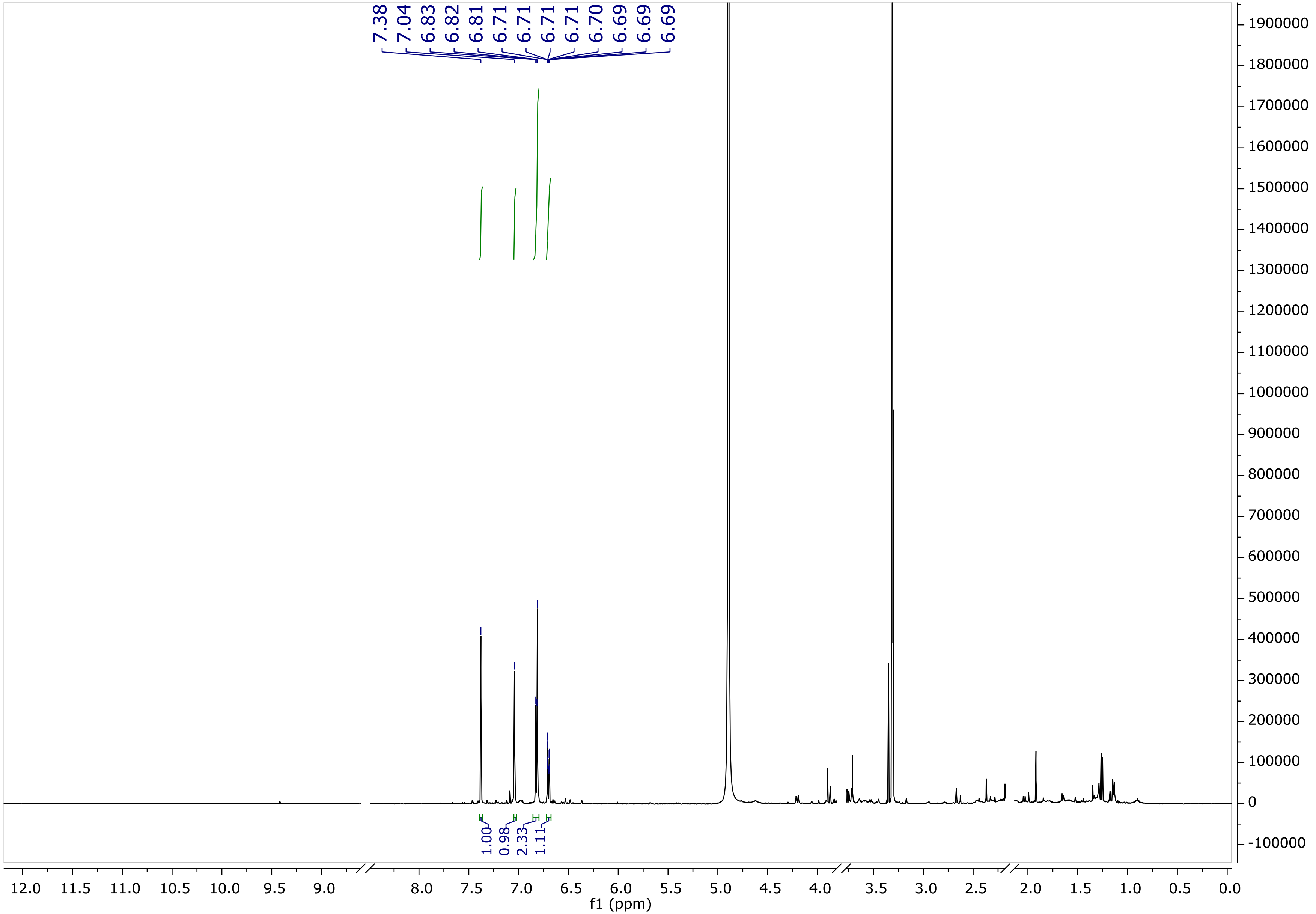

Figure S4. ^{1}H NMR spectrum of **1** in methanol-d_4 at 500 MHz.

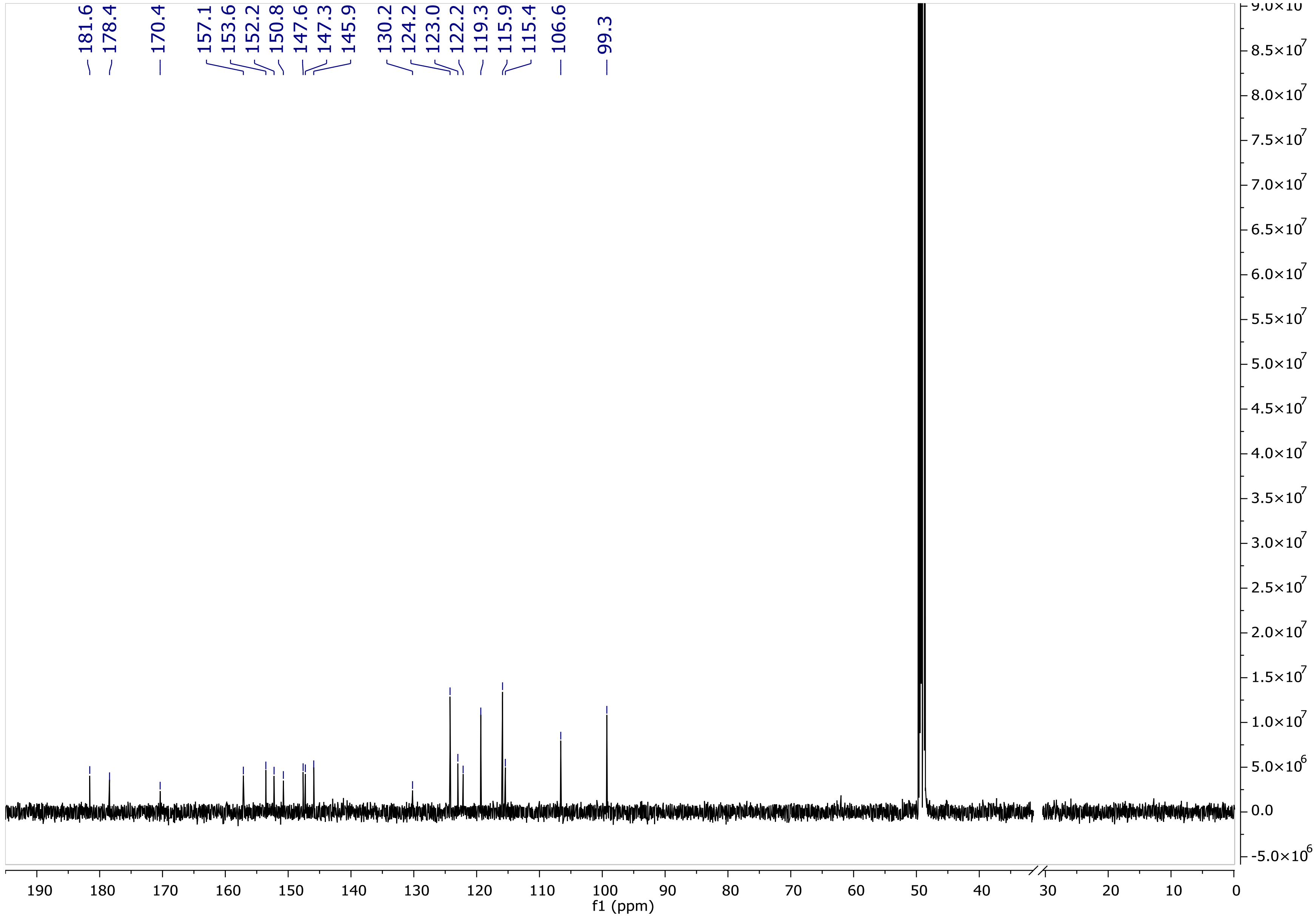

Figure S5. ^{13}C NMR spectrum of **1** in methanol-d_4 at 125 MHz.

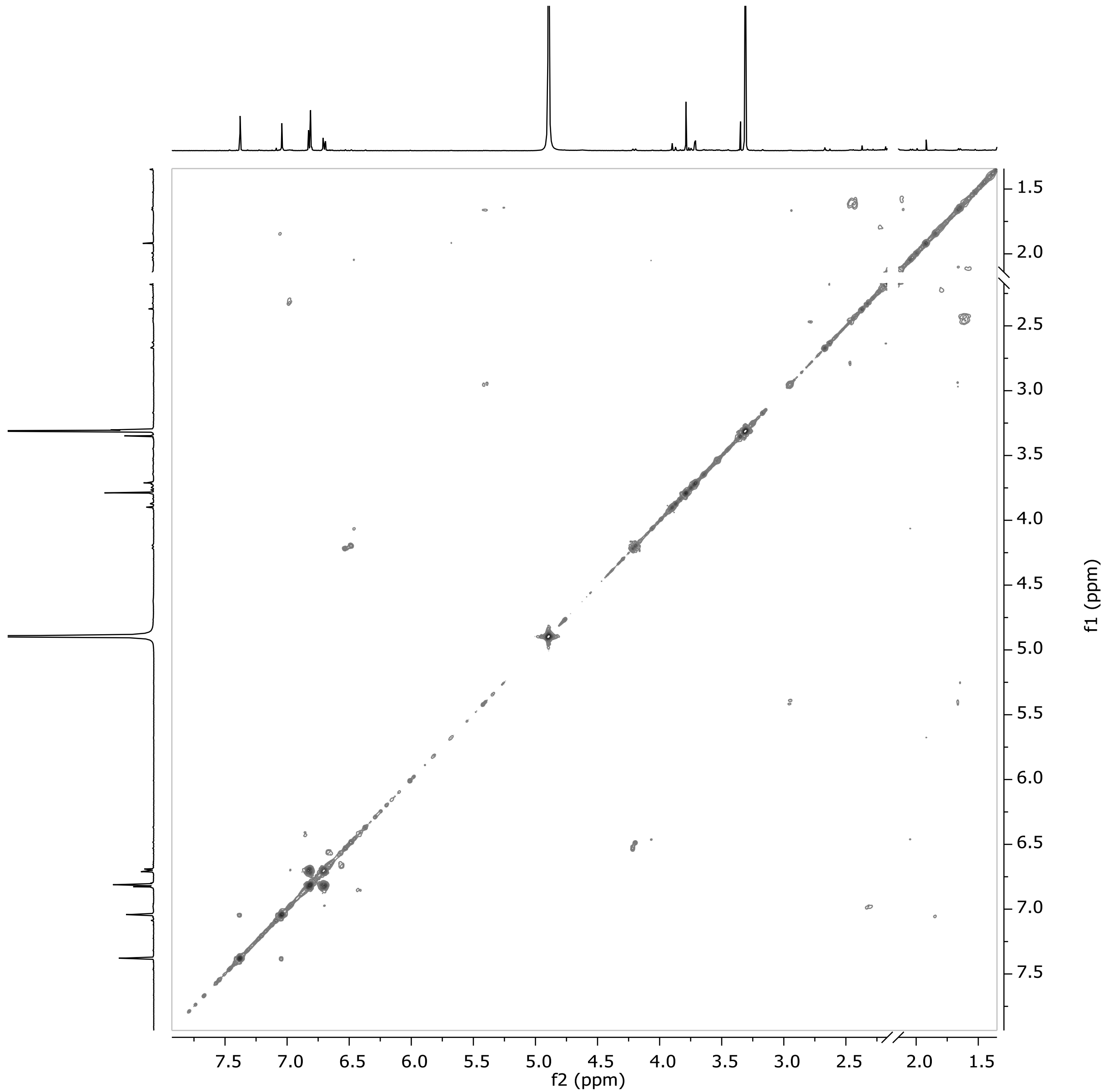

Figure S6. ^{1}H–^{1}H NMR spectrum of **1** in methanol-d_4 at 500 MHz.

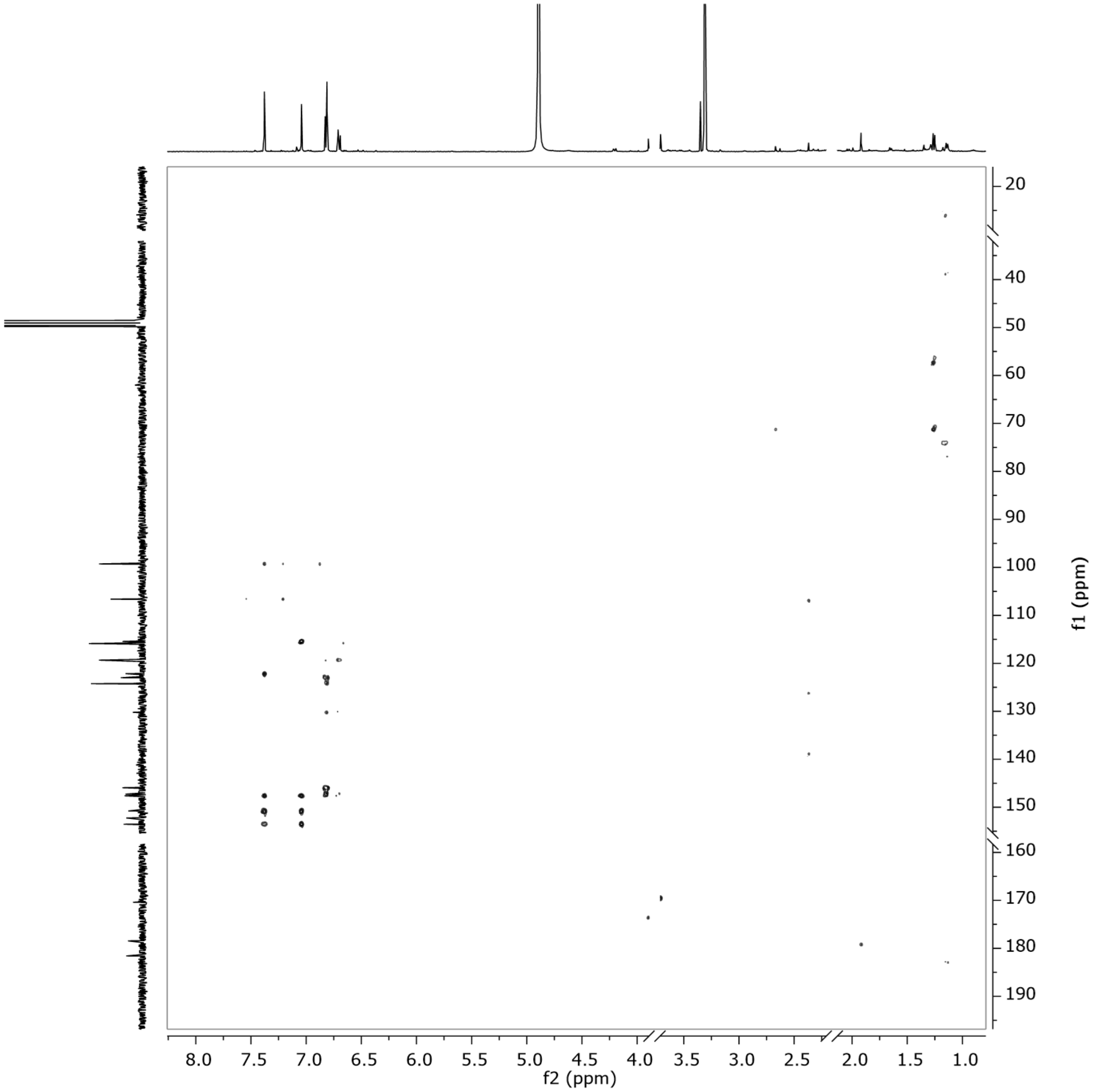

Figure S7. HMBC spectrum of **1** in methanol-d_4 at 500 MHz.

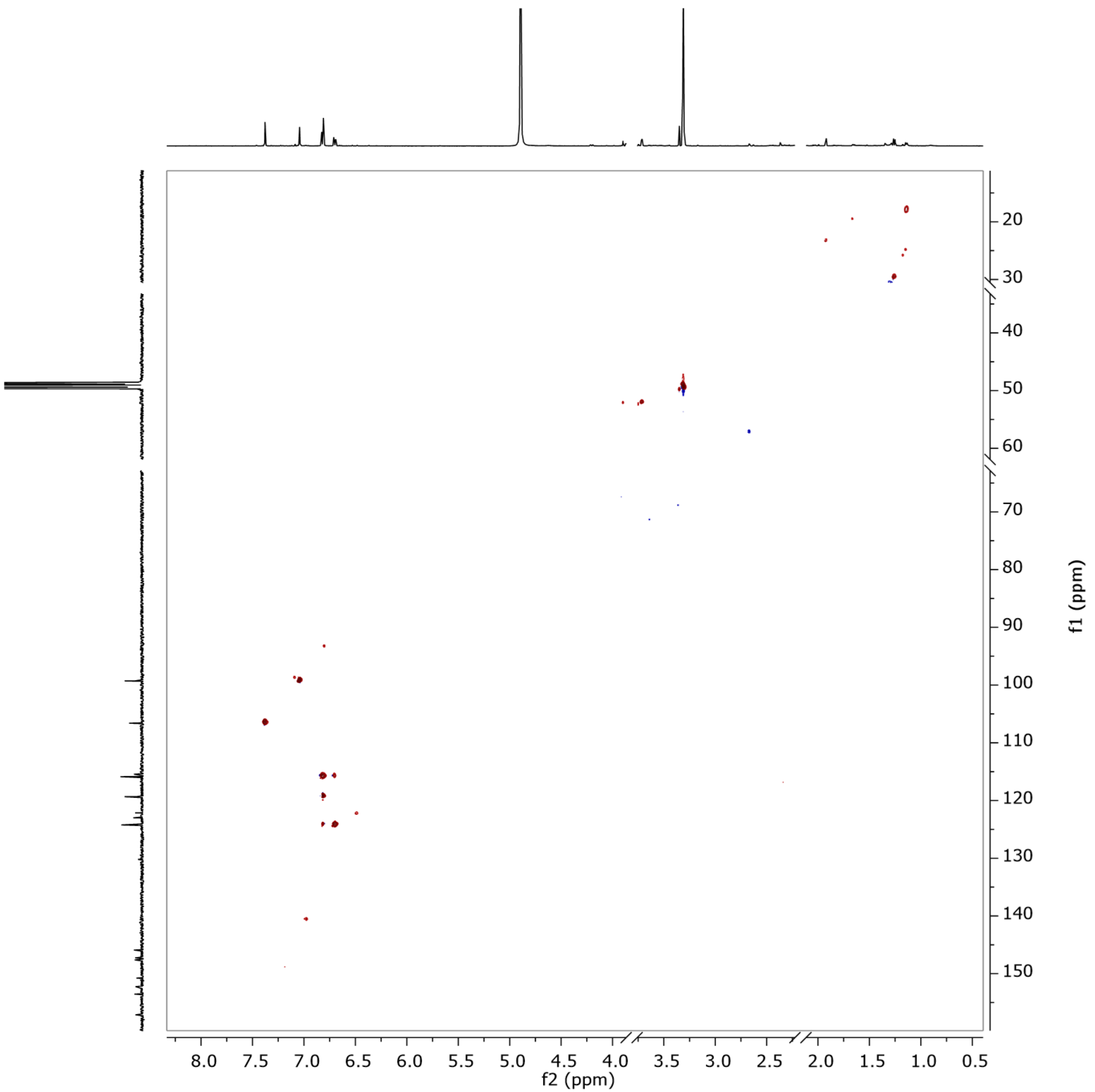

Figure S8. HSQC spectrum of **1** in methanol-d_4 at 500 MHz.

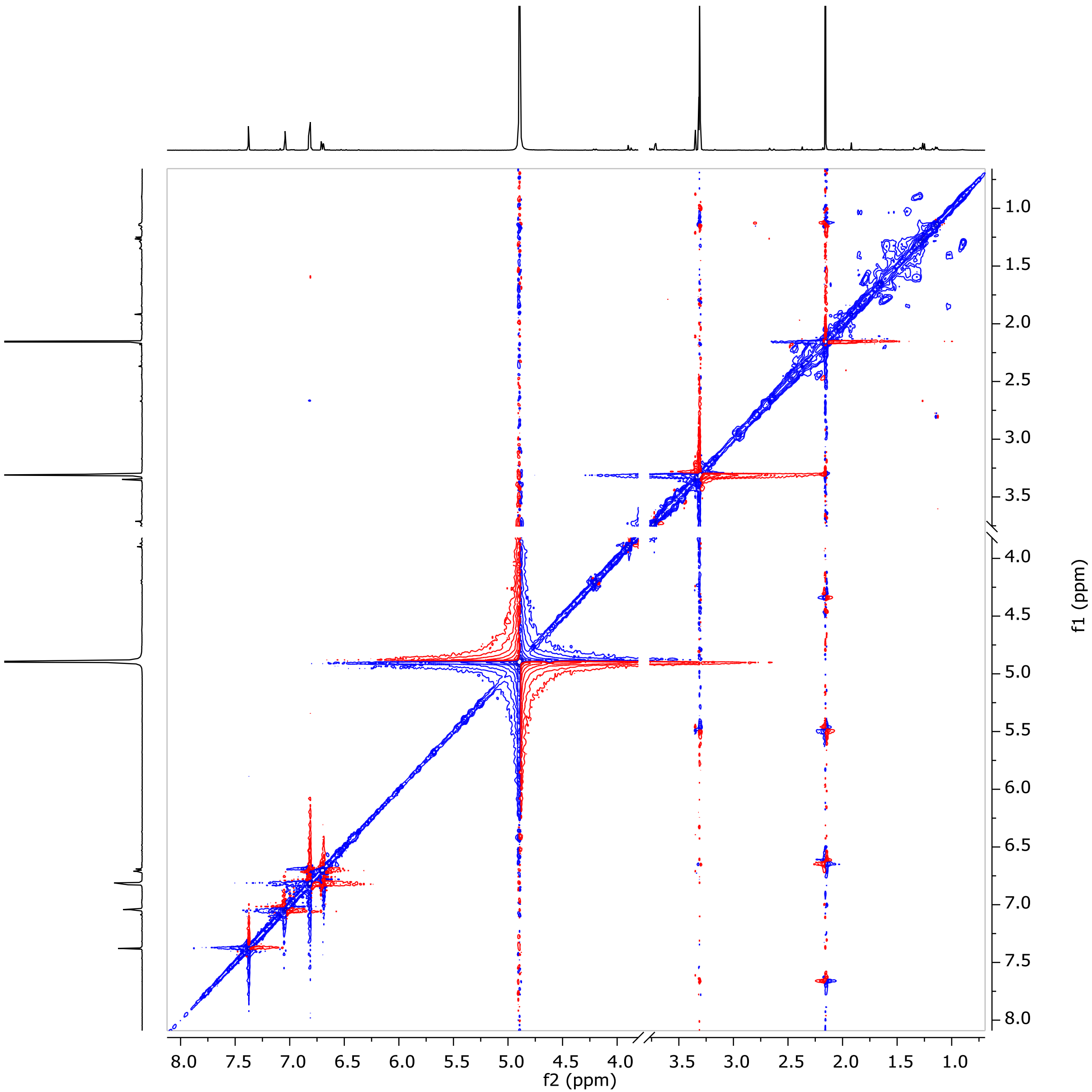

Figure S9. ROESY spectrum of **1** in methanol-d_4 at 500 MHz.

Display Report

Analysis Info

Analysis Name	S:\DATA\AmaZon\wsu20_Winnier Sum Chemutai\IHI CRUDE\IHI 752\752 PURE COMPOUNDS\IHI 752B R2F1_BC1_01_47335.d	Acquisition Date	22.05.2023 16:58:12
Method	47335.m	Operator	tti
Sample Name	IHI 752B R2F1	Instrument	amaZon speed
Comment			

Acquisition Parameter

Ion Source Type	ESI	Ion Polarity	Positive	Alternating Ion Polarity	on
Mass Range Mode	UltraScan	Scan Begin	100 m/z	Scan End	2000 m/z
Accumulation Time	4000 µs	RF Level	100 %	Trap Drive	68.9
SPS Target Mass	1000 m/z	Averages	6 Spectra		

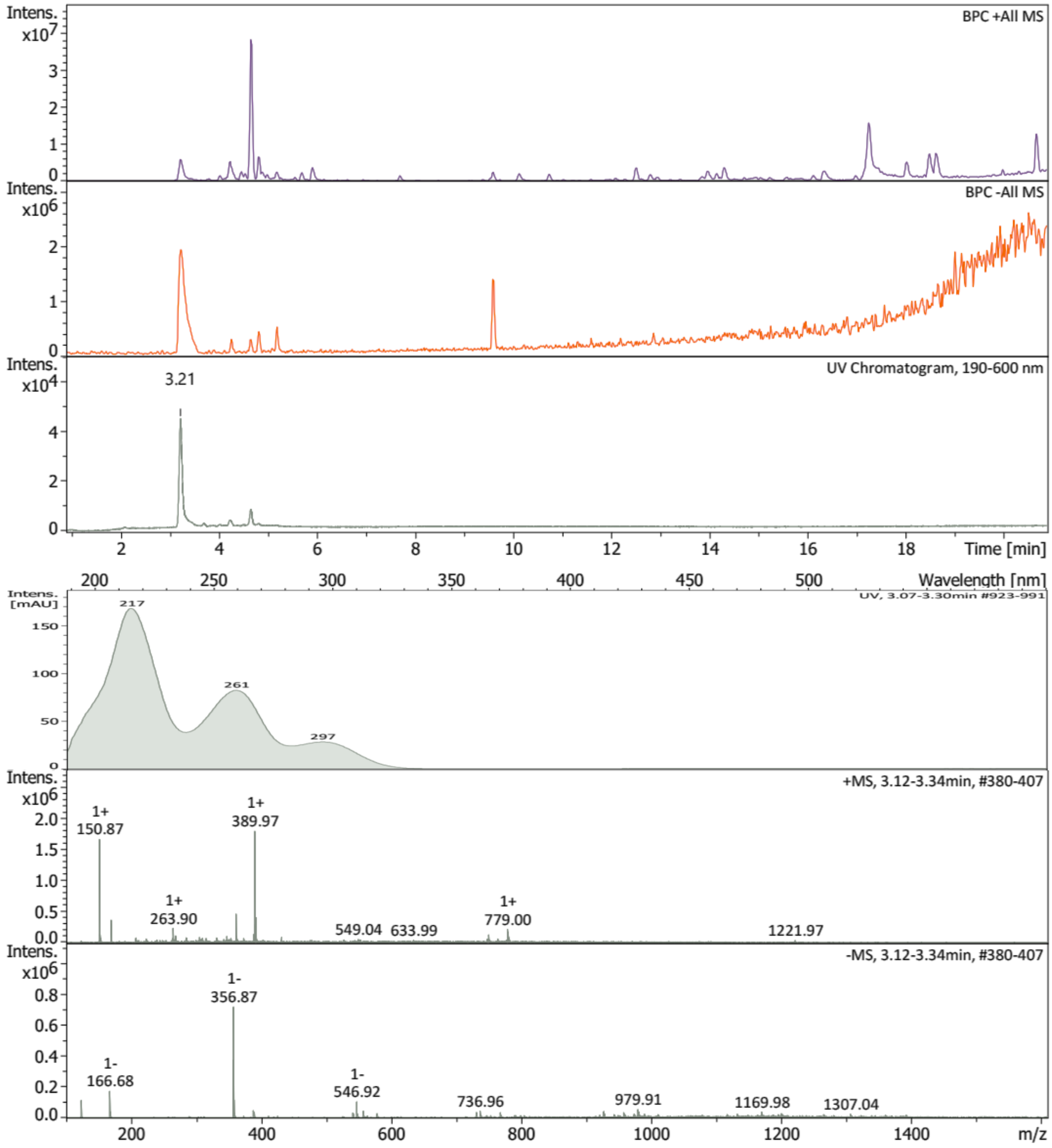

Bruker Compass DataAnalysis 6.1 printed: 27.03.2024 15:43:49 by: sel22 Page 1 of 1

Figure S10. LR-ESI-MS of **2**.

Display Report

Analysis Info

		Acquisition Date	23.05.2023 18:58:34
Analysis Name	S:\DATA\MaXis\wsu20_Winnie Sum Chemutai\23_05\IHI 752B R2F1_25_01_11588.d		
Method	pos_säure_10000_screening_ms_100_2500_line.m	Operator	ate06
Sample Name	IHI 752B R2F1	Instrument	maXis
Comment	Screening01<br>Waters Acquity UPLC BEH C_18 1,7um 2.1x50mm		

Acquisition Parameter

Ion Polarity Positive

SPS Target Mass

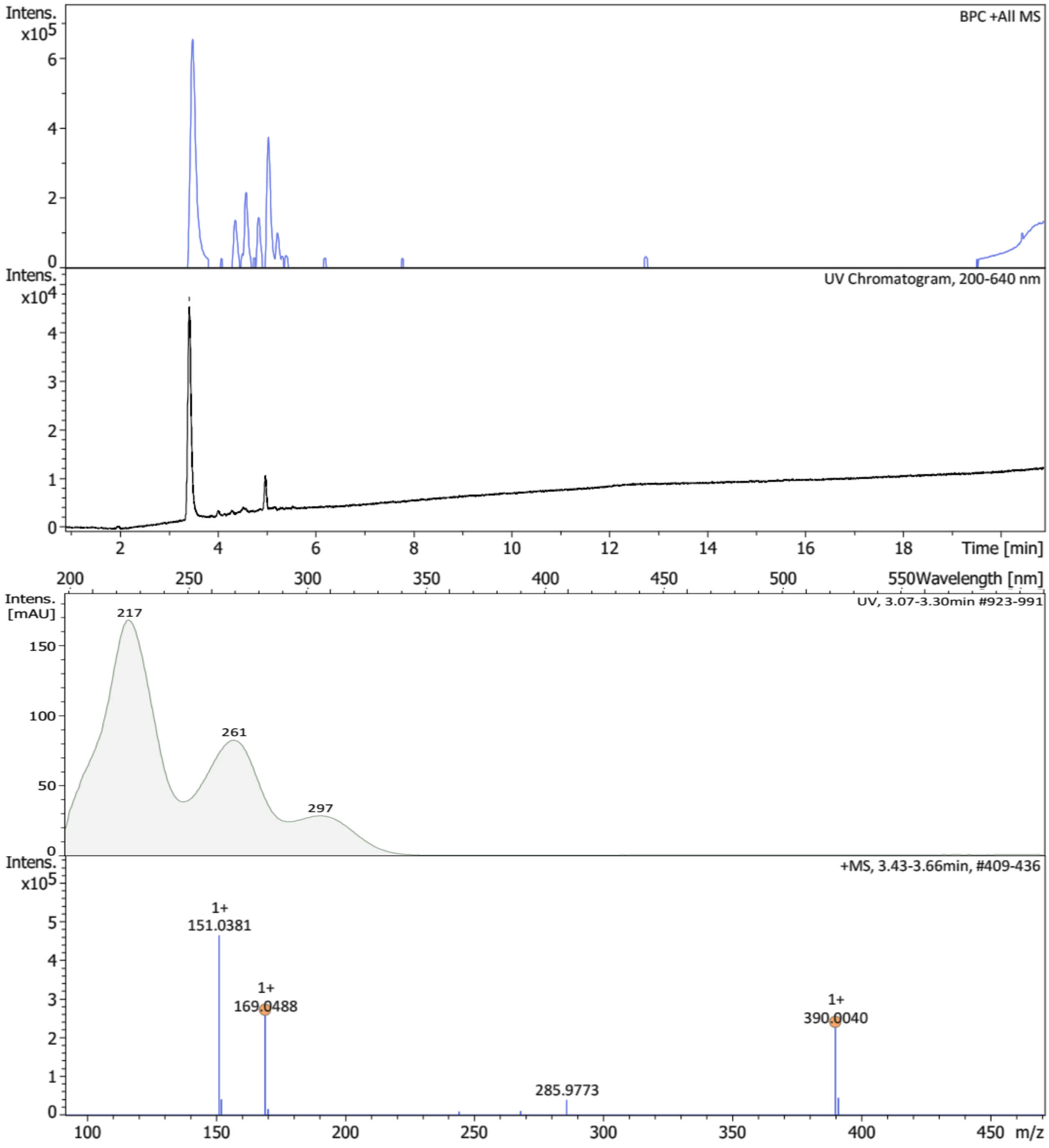

Bruker Compass DataAnalysis 6.1 printed: 27.03.2024 15:49:31 by: sel22 Page 1 of 1

Figure S11. HR-ESI-MS of **2**.

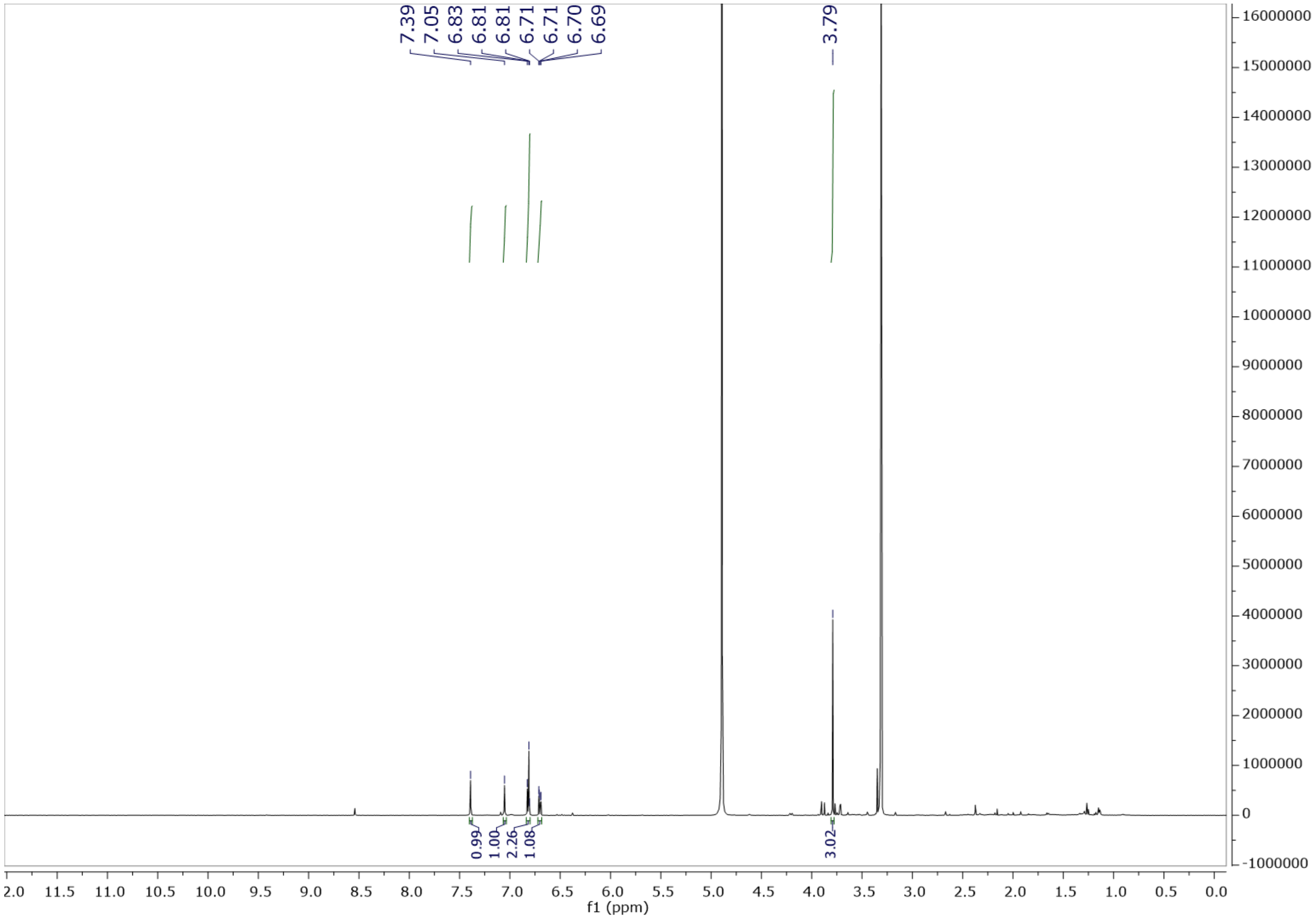

Figure S12. ^{1}H NMR spectrum of **2** in methanol-d_4 at 500 MHz.

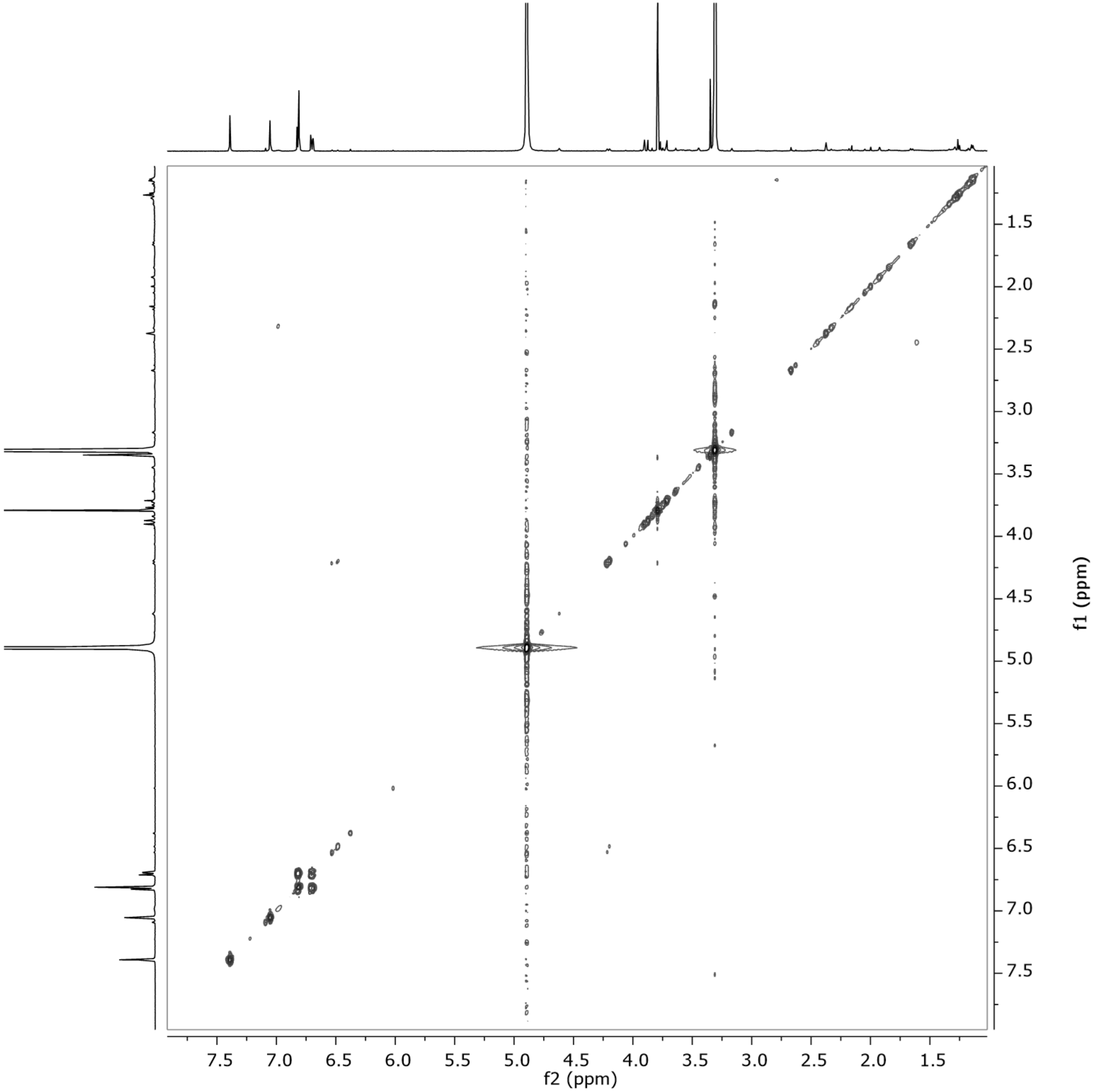

Figure S13. ^{1}H-^{1}H COSY spectrum of **2** in methanol-d_4 at 500 MHz.

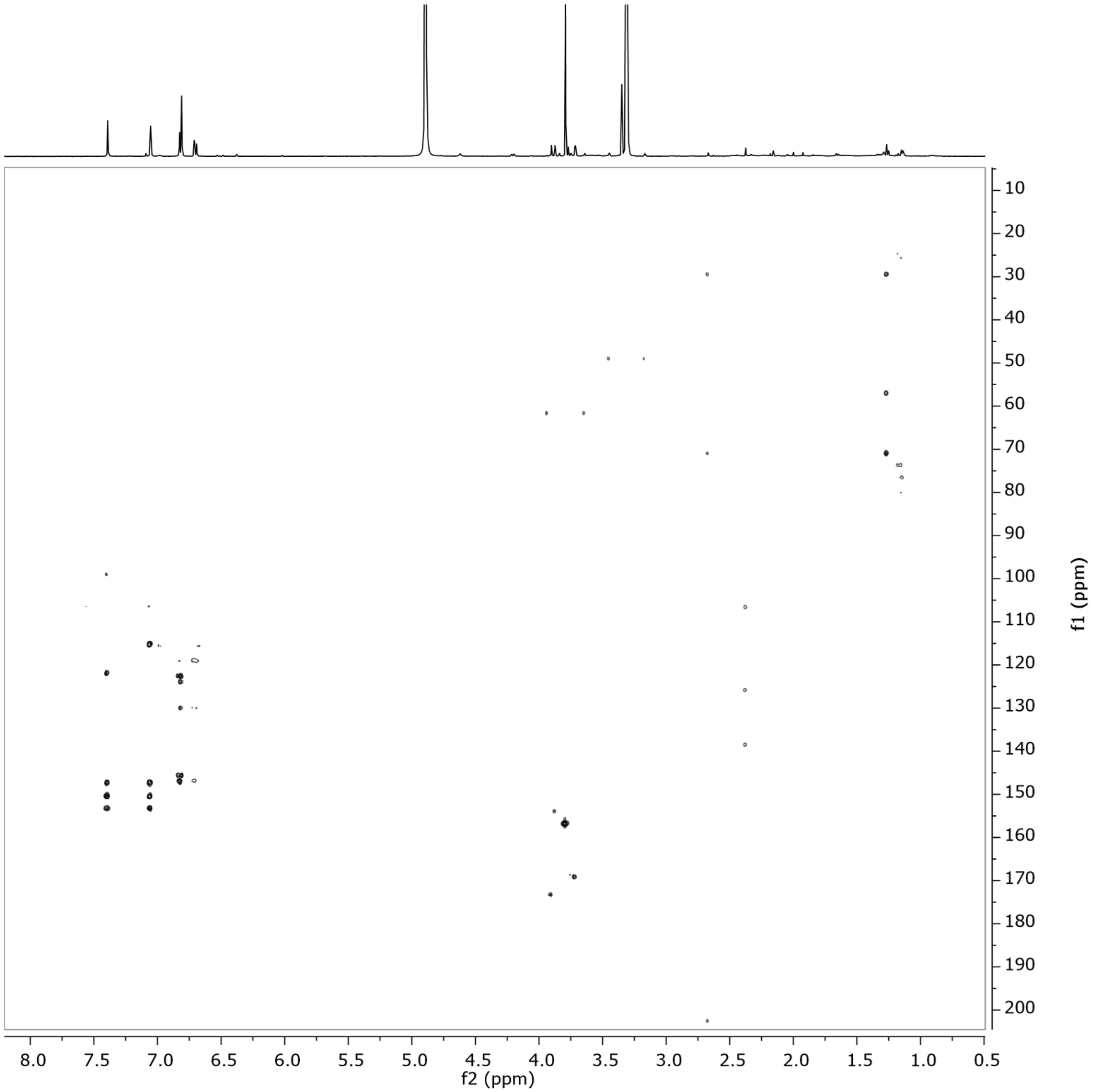

Figure S14. HMBC spectrum of **2** in methanol-d_4 at 500 MHz.

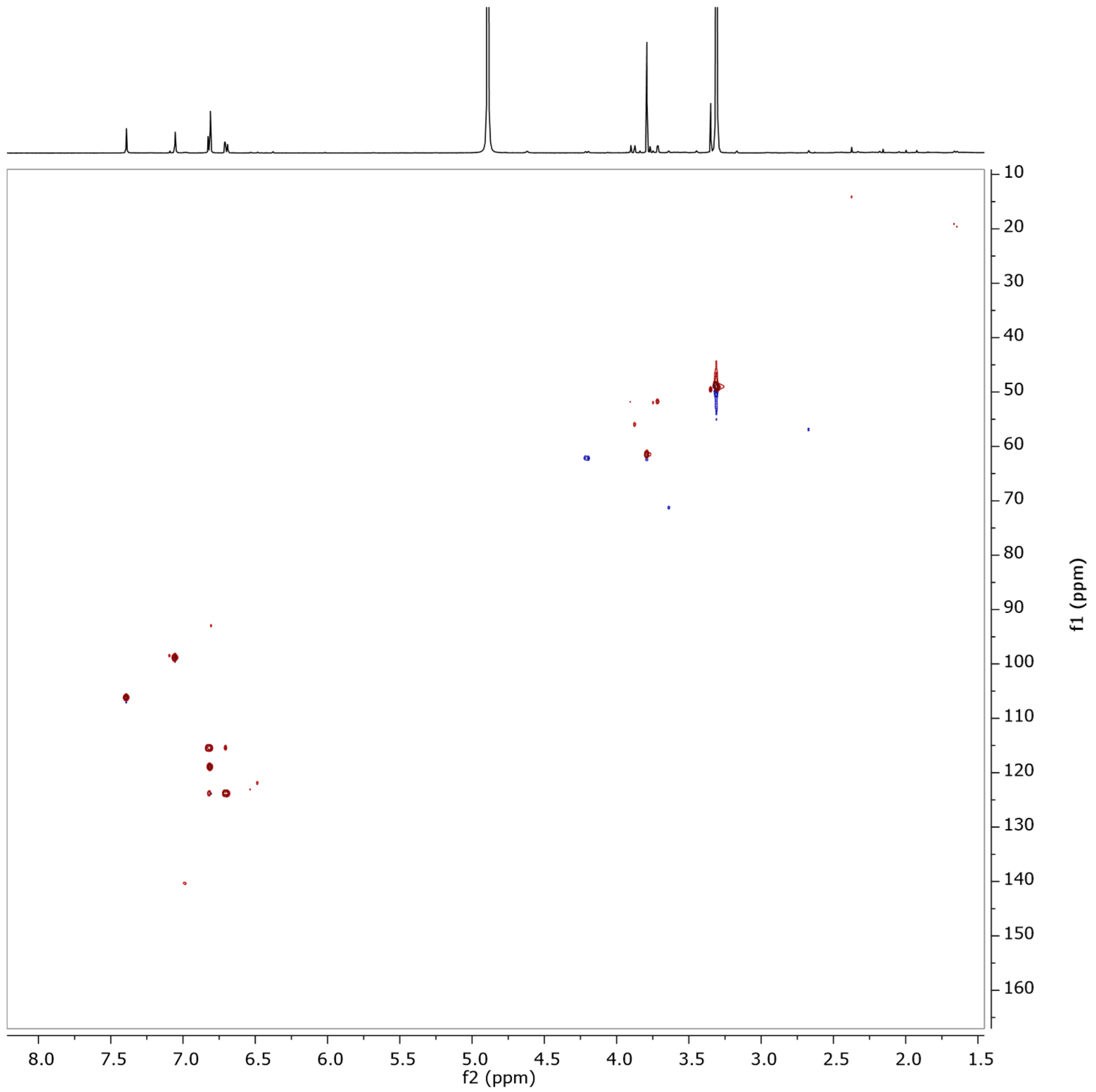

Figure S15. HSQC spectrum of **2** in methanol-d_4 at 500 MHz.

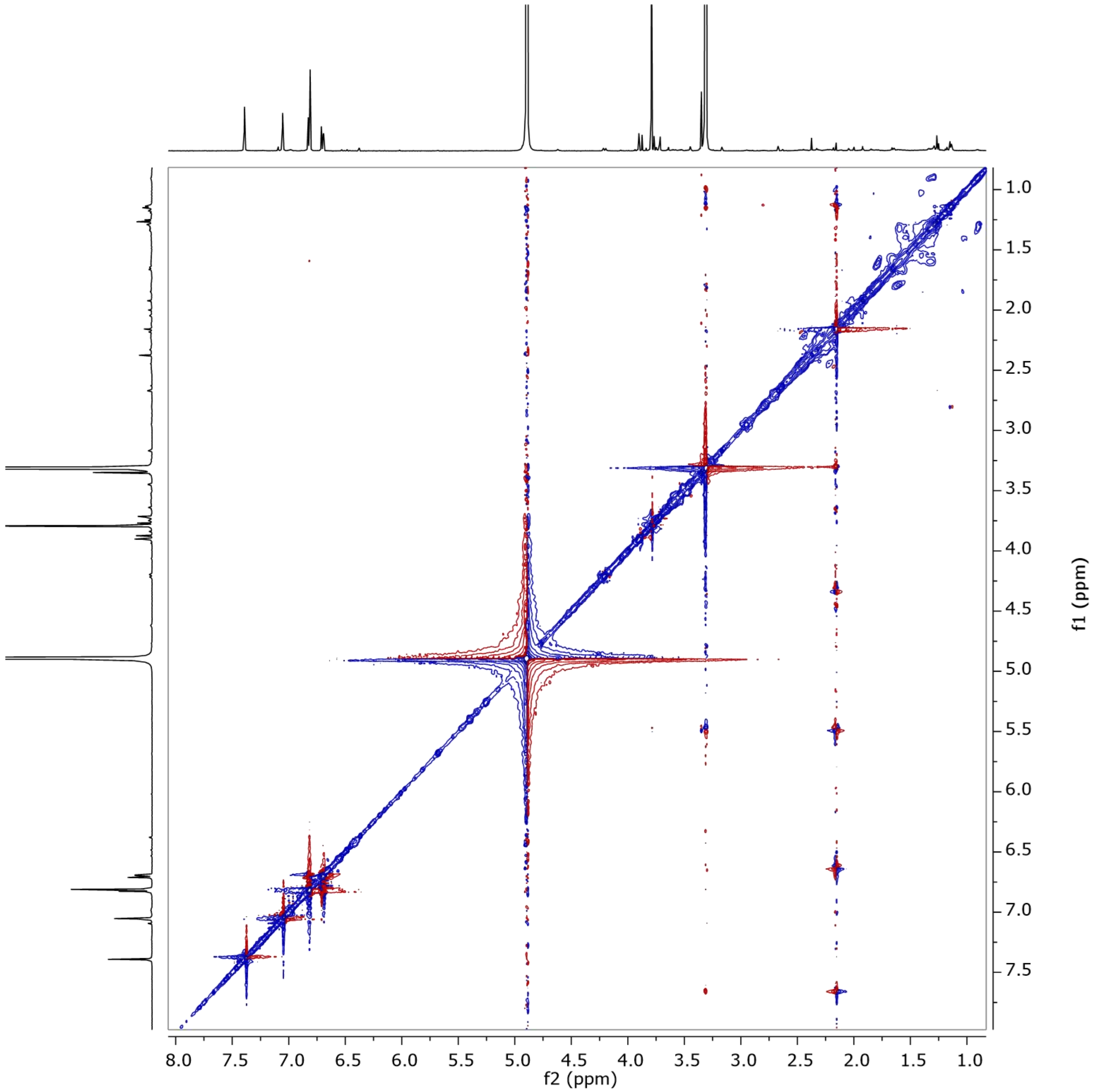

Figure S16. ROESY spectrum of **2** in methanol-d_4 at 500 MH

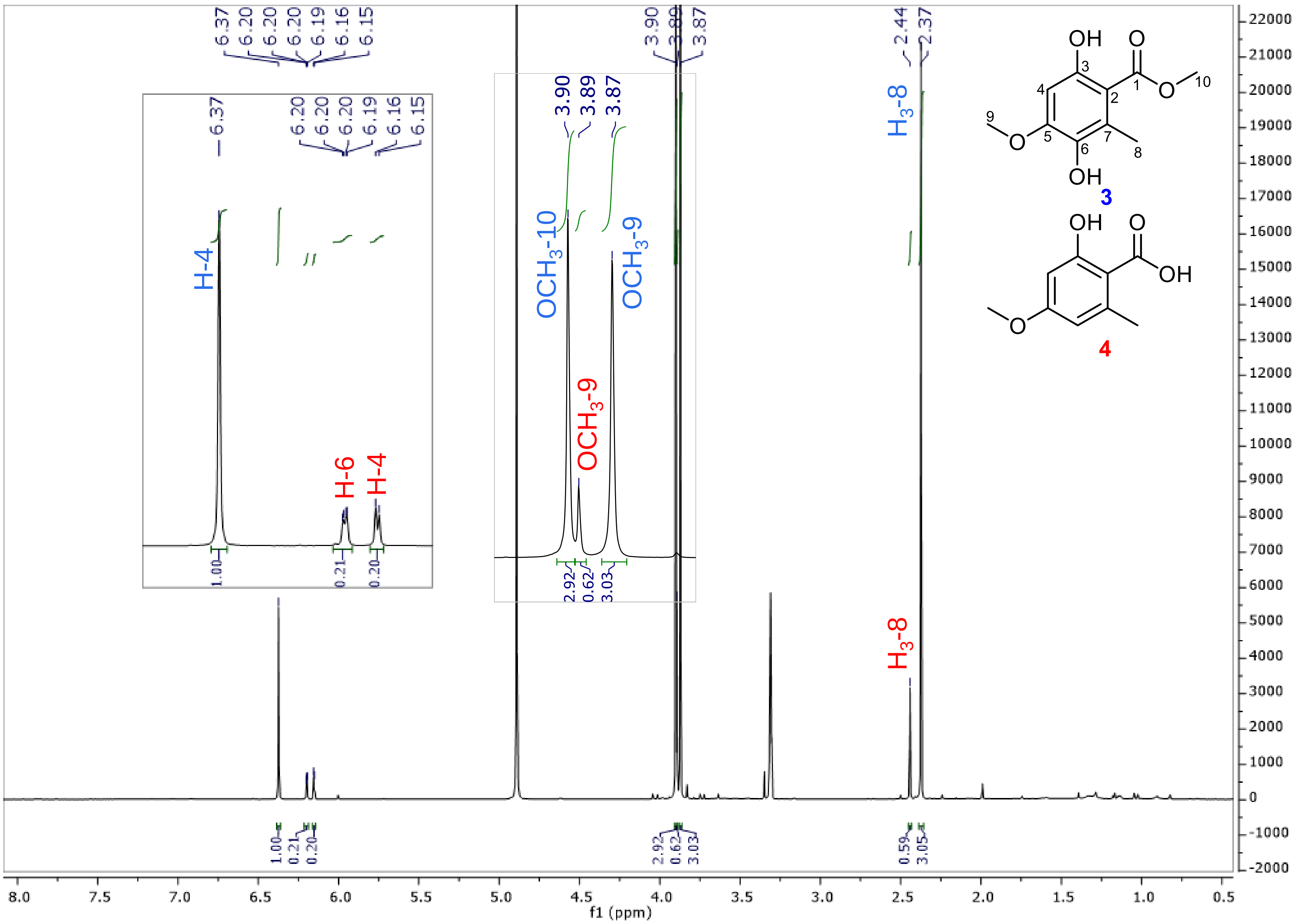

Figure S17. ^{1}H NMR spectrum of **3** and **4** in methanol-d_4 at 500 MHz.

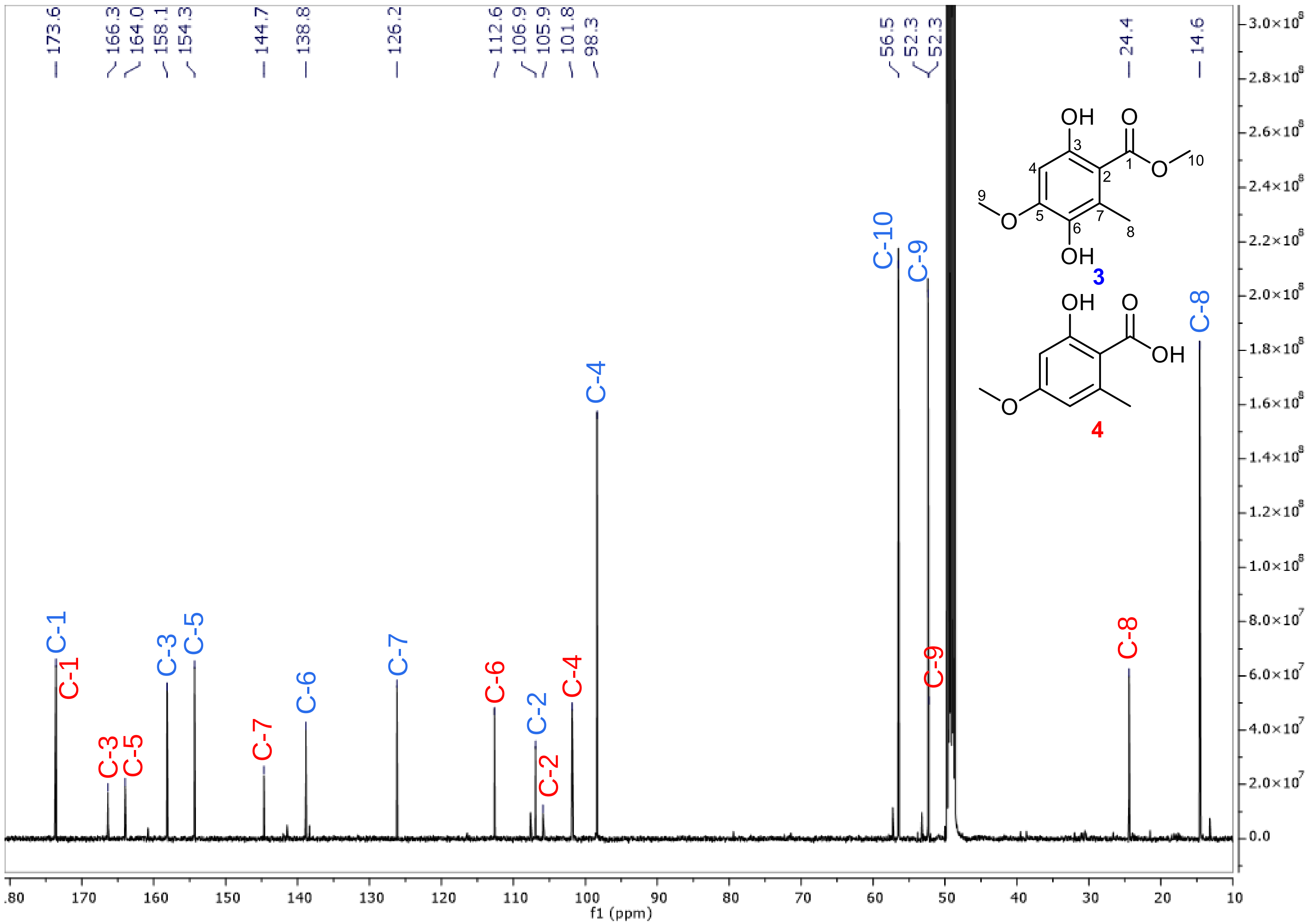

Figure S18. ^{13}C NMR spectrum of **3** and **4** in methanol-d_4 at 125 MHz.

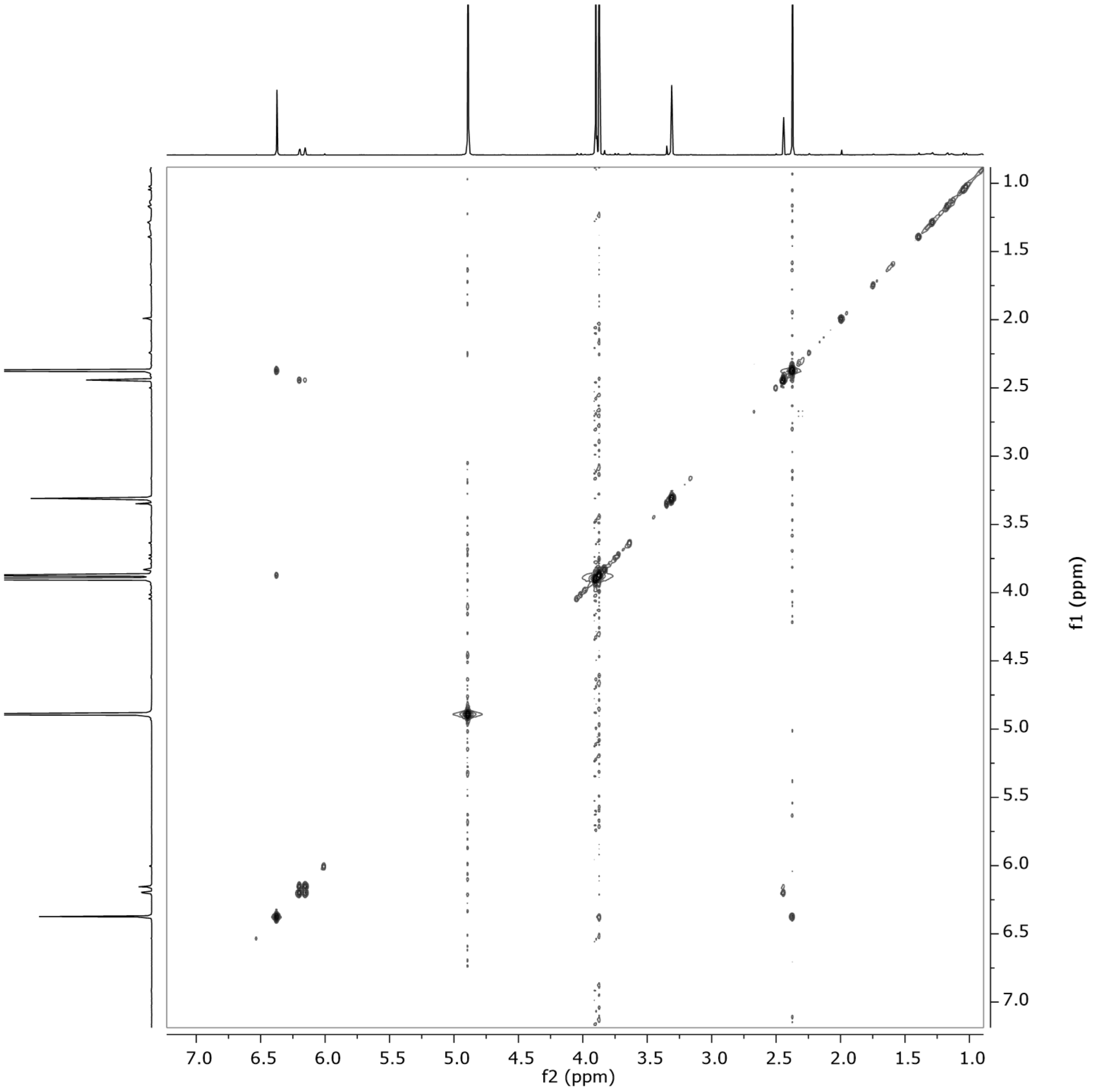

Figure S19. ^{1}H-^{1}H COSY spectrum of **3** and **4** in methanol-d_4 at 500 MHz.

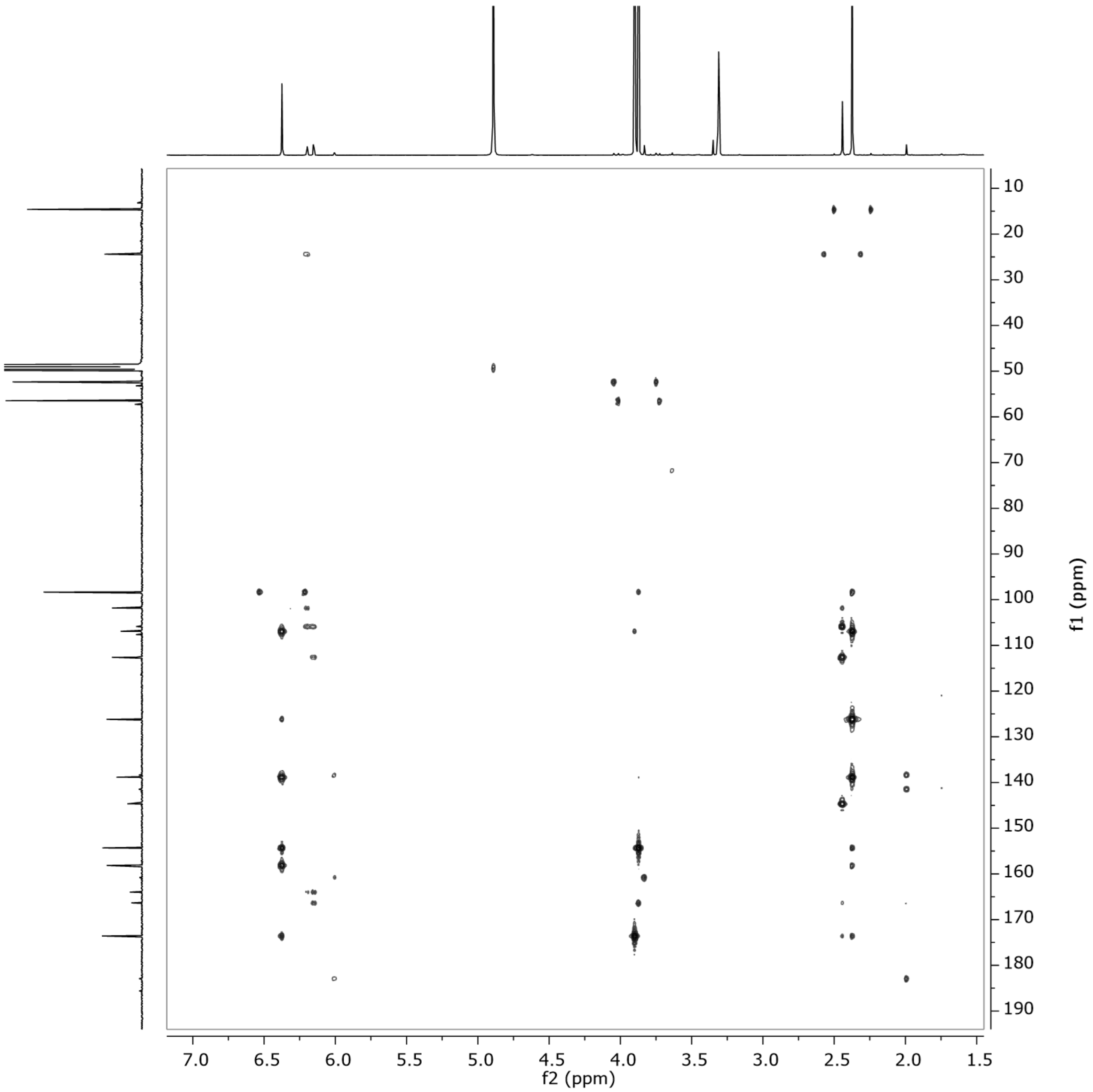

Figure S20. HMBC spectrum of **3** and **4** in methanol-d_4 at 500 MHz.

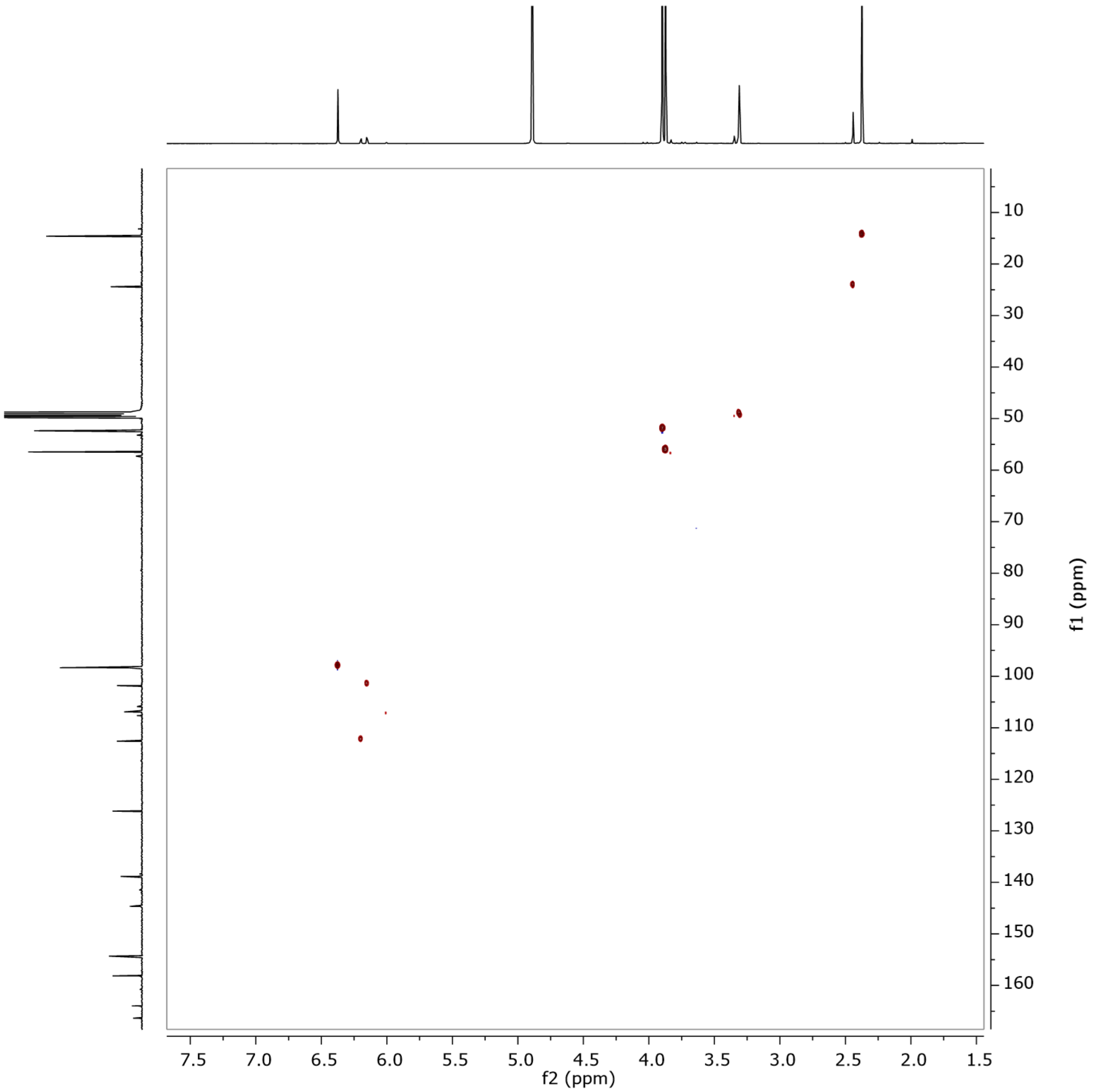

Figure S21. HSQC spectrum of **3** and **4** in methanol-d_4 at 500 MHz.

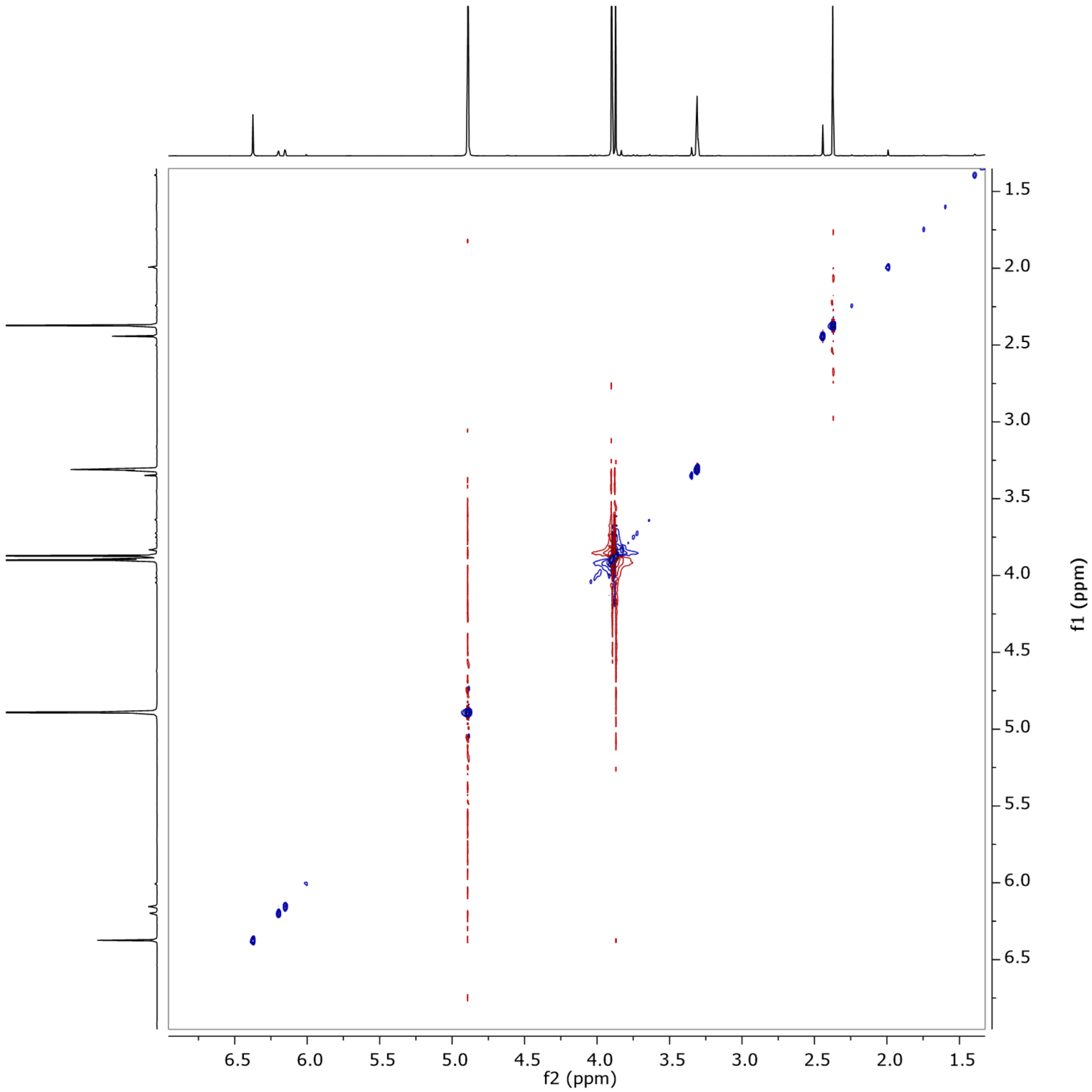

Figure S22. ROESY spectrum of **3** and **4** in methanol-d_4 at 500 MHz.
